# The geometric and dosimetric accuracy of kilovoltage cone beam computed tomography images for adaptive treatment: a systematic review

**DOI:** 10.1259/bjro.20220062

**Published:** 2023-05-16

**Authors:** Hussam Jassim, Hassan A. Nedaei, Ghazale Geraily, Nooshin Banaee, Ali Kazemian

**Affiliations:** 1 Department of Medical Physics, Tehran University of Medical Sciences, Tehran, Iran; 2 Radiation Oncology Research Centre, Cancer Institute, Tehran University of Medical Sciences, Tehran, Iran; 3 Radiotherapy Department, National Cancer Hospital, Kufa, Iraq; 4 Medical Radiation Research Center, Islamic Azad University, Tehran, Iran

## Abstract

**Objectives:**

To provide an overview and meta-analysis of different techniques adopted to accomplish kVCBCT for dose calculation and automated segmentation.

**Methods:**

A systematic review and meta-analysis were performed on eligible studies demonstrating kVCBCT-based dose calculation and automated contouring of different tumor features. Meta-analysis of the performance was accomplished on the reported γ analysis and dice similarity coefficient (DSC) score of both collected results as three subgroups (head and neck, chest, and abdomen).

**Results:**

After the literature scrutinization (*n* = 1008), 52 papers were recognized for the systematic review. Nine studies of dosimtric studies and eleven studies of geometric analysis were suitable for inclusion in meta-analysis. Using kVCBCT for treatment replanning depends on a method used. Deformable Image Registration (DIR) methods yielded small dosimetric error (≤2%), γ pass rate (≥90%) and DSC (≥0.8). Hounsfield Unit (HU) override and calibration curve-based methods also achieved satisfactory yielded small dosimetric error (≤2%) and γ pass rate ((≥90%), but they are prone to error due to their sensitivity to a vendor-specific variation in kVCBCT image quality.

**Conclusions:**

Large cohorts of patients ought to be undertaken to validate methods achieving low levels of dosimetric and geometric errors. Quality guidelines should be established when reporting on kVCBCT, which include agreed metrics for reporting on the quality of corrected kVCBCT and defines protocols of new site-specific standardized imaging used when obtaining kVCBCT images for adaptive radiotherapy.

**Advances in knowledge:**

This review gives useful knowledge about methods making kVCBCT feasible for kVCBCT-based adaptive radiotherapy, simplifying patient pathway and reducing concomitant imaging dose to the patient.

## Introduction

Kilovoltage Cone Beam Computed Tomography (kVCBCT) is imaging system that uses cone beam computed tomography methods to achieve tomographic imaging. kVCBCT started to be used for patient setup verification in the mid-2000 when Jaffray et al.^1^ began researching on-gantry implementation for radiation therapy guidance.^1^ The latest On-gantry kVCBCT systems involve a kV source and large-area flat-panel detectors attached to the linac gantry, often orthogonally to the treatment beam. The path of source-digital detector is circular arc (a half-scan) or orbit (full-scan). On-gantry kVCBCT systems commercially available are: the X-ray Volume Imager (XVI) by Elekta, On-Board Imager (OBI) by Varian Medical Systems, Vero by (BrainLAB AG, Feldkirchen, Germany & MHI, Mitsubishi Heavy Industries, Japan), and the kVision by Siemens.^2^

kVCBCT is utilized to correct the daily treatment errors. Among the daily treatment errors are setup errors, errors due to the complexity of the MLC delivery, internal organ motion, weight loss, and structural deformation.^3–5^ Mendes et al.^6^ and Roeske et al.^7^ showed a 10% variation in prostate volumes occurs during radiation therapy.^6,7^ If an error is made, the therapeutic ratio will become worsened; therefore, cure rate will be reduced, radiation side-effects will increase, or the patient even can die from excessive radiation exposure. An important strategy for reducing this error is recognizing on a daily basis the location of a tumor relative to healthy tissue. The uses of kVCBCT attached to the linac as “Image Guided Radiation Therapy” are robust methods for evaluating inter- and intrafraction deformation. Using this technology, the shift or volume deformation of the tumor can be corrected prior to radiation delivery.

Although kVCBCT is employed as an image guidance technology to minimize geometric uncertainty, it is expected that the actual dose to the target and organs at risk (OARs) may differ from the estimated values due to multiple factors, including weight loss, motion during interfractions, tumor regression, and progression, as well as organ deformation. To better understand the dose-response relationship to further improve the therapeutic ratio, more attention has being paid to calculate the dose of the day to increase the precision of the actual dose delivery. The paradigm for radiotherapy has shifted from image guidance to dose guidance. On a practical level, using kVCBCT for online planning to deliver palliative radiotherapy has been proven effective, with an adequate dosimetric accuracy and planning time.^8^ As such, it can be used for both verification parameters, such as patient anatomy and set-up, and online dose calculation. kVCBCT image for dose calculation enables physicists to verify the actual distribution of dose on any given treatment day. Nevertheless, kVCBCT systems are limited in their ability to perform dosimetric verification in curative radiotherapy. In other words, Hounsfield Unit (HU) values are inherently unreliable for dose calculations for many reasons. First of all, high scatter photons participate in the transmitted projections by virtue of a large irradiated volume. Secondly, the relationship between HUs and attenuation coefficient (µelec) may differ for different patients and organs of the same patient. Finally, acquisition parameters, for example the number of projections, limited gantry rotation speed, tube current and potential, cause the degradation of the image quality.^4,8–10^ As a result, kVCBCT images cannot be used directly to calculate dose distribution.

In cases where kVCBCT images demonstrate significant anatomical deformations, only using another standard CT imaging can provide a reliable dose distribution measurement. In fact, this procedure is labor-intensive and gives additional dosage to the patients in terms of concomitant imaging dose.^11^ It has been proposed many methods for converting kVCBCT into computed tomography (CT)-equivalent representations, commonly called synthesized CT, that can be used for dose calculation, treatment planning, and adaptive radiotherapy. Also, many of these methods could be used for propagating contours on planning CT (pCT) images into CT-equivalent kVCBCT or kVCBCT for different parts of the body.^12,13^

In spite of large number of scientific literatures covering this subject, an inclusive overview and meta-analysis of the geometric and dosimetric accuracy of kVCBCT images for adaptive treatment is still lacking. Therefore, there are illusion about how kVCBCT can be used for adaptive radiotherapy. The aim of the current review hence was to provide a systematic review and meta-analysis of the geometric and dosimetric accuracy of kVCBCT for treatment replanning. By providing a set of recommendations for reporting future kVCBCT dosimetric and geometric studies, the advantages and disadvantages of the techniques reported are critically assessed by evaluating metrics for dosimetric and geometric analysis, feasibility, and reproducibility.

### The process of both offline and online image review

“Adaptive Radiation Therapy (ART) is a closed-loop radiation treatment process, where the treatment plan can be modified using systematic feedback of delivered dose information. It intends to improve radiation treatments by monitoring treatment variations and incorporating them into reoptimization of the treatment plan”.^14^ ART can be classified into Offline or Online ART depending on a particular selection of the ART timescale. Whilst Offline ART is performed by imaging the patient in time interval between fractions, Online ART is performed immediately prior the treatment fraction. The former uses workflow and tools of conventional treatment planning, which makes it requiring few specialized tools; however, interfraction anatomical variations might take place when using Offline ART that will lead to inducing of further geometric error. The latter uses integrated tools to the delivery control system. Therefore, the problem of interfraction anatomical variations can be solved when using Offline ART. Online ART also faces some challenges, for instance large intrafractional variations and lack of measurements availability of patient-specific quality assurance.^15^

### Description of adaptive pathways

Immediately prior to a treatment fraction, kVCBCT system integrated to the linac is used to acquire kVCBCT images. These images are registered with pCT to verify the patient setup. The registered image represents the starting point for ART when at this point radiotherapy professionals decide whether the original plan needs to be modified in case of structural differences between pCT and kVCBCT. When there is identification of the clinical need for adaptation, a new physician orders and radiation therapy prescription, new simulation, new treatment planning, new localization, new imaging, new assessment, new replanning, new quality assurance and new delivery are introduced.^15^ In this situation, kVCBCT images would reduce further stressors on patients with mobility issues, avoid delaying a treatment start for new patient by an additional CT simulation time slot in a busy radiation oncology center, radiation exposure for patients and cost if and only if possibility of kVCBCT-based dose calculation is performed.

### kVCBCT treatment replanning methodology

Several strategies have been employed for correcting kVCBCT HU for dose calculation and propagating contours from pCT images into kVCBCT. The main categories of approaches used for dose computation are deformable image registration (DIR), deep learning algorithm, calibration curve, and density override, combined and hybrid algorithm. For contours propagations, on the other hand,DIR and deep learning algorithm were used.

#### kVCBCT for dose computation

Calculating dose with kVCBCT system is not very accurate because HU values are inherently unreliable when calculating dose. Numerous factors make HUs inherently unreliable for dose calculations. Firstly, the kVCBCT HU sensitivity increases as the object size increases since large irradiated volume contributes to a large scattering of photons in transmitted projections. Furthermore, a patient’s HUs and µelec may differ depending on the organ they belong to. Finally, acquisition parameters, such as the number of projections, the rotation speed of the gantry, tube currents and potentials can degrade the image quality.^9,10,16,17^ In the process of correcting kVCBCT HU for dose calculation, many correction strategies have been used as described below.

#### Deformable image registration

The process of image registration involves aligning images in order to connect corresponding features. A registration is achieved by modifying an image by altering its geometry and intensity through geometric operations. An image is registered by aligning features with physical locations or with computer simulations. Alternatively, the images might have been collected with the corresponding sensor at different intervals or with a variety of sensors; for instance, each sensor may be sensitive to a distinct part of the electromagnetic spectrum.

A geometric operation converts a given image I into a new image I **'** by modifying the pixels' coordinates. By assuming the intensities of the image are unchanged but their positions do, this means the image function value ( I ) at the original location (x, y) is transferred to the new location (x**^'^**, y**^'^**) in the transformed image I **^'^** by applying the transformation function (T) that is required to model this process. This section discusses image registration applications used in medical imaging. This covers a variety of image usage but focuses primarily on imaging used in in room radiotherapy.

In image registration, the similarity between images is evaluated using complex methods, including cues and content, to identify similarities between images. There are three methods of DIR, which are physical model methods, model-based methods and hybrid methods. While physical model methods include intensity-based methods and Biomechanical-Based Modelling, model-based methods include point-based methods, and hybrid method.^12,18,19^ The next subsections deal with intensity-based methods, point-based methods, biomechanical-based modeling and hybrid methods that use one method as the first condition to another one.

##### Intensity-based methods

A method based on intensity values operates on the intensity values of the whole image content without removing any features first. A smooth transformation is always searched for that maximizes the measure of intensity-based similarity. The problem with these methods is that, despite the fact that they can act without user interaction, they have high computational costs and require preregistration to register because the two source images should be close enough.^15,20,20,21^ Intensity-based methods can be classified into optical flow**,** demon’s algorithm, and level-set algorithm. for more details, see (Yang et al. (2011) and references therein.^22^

###### Optical flow

**Figure 1.**
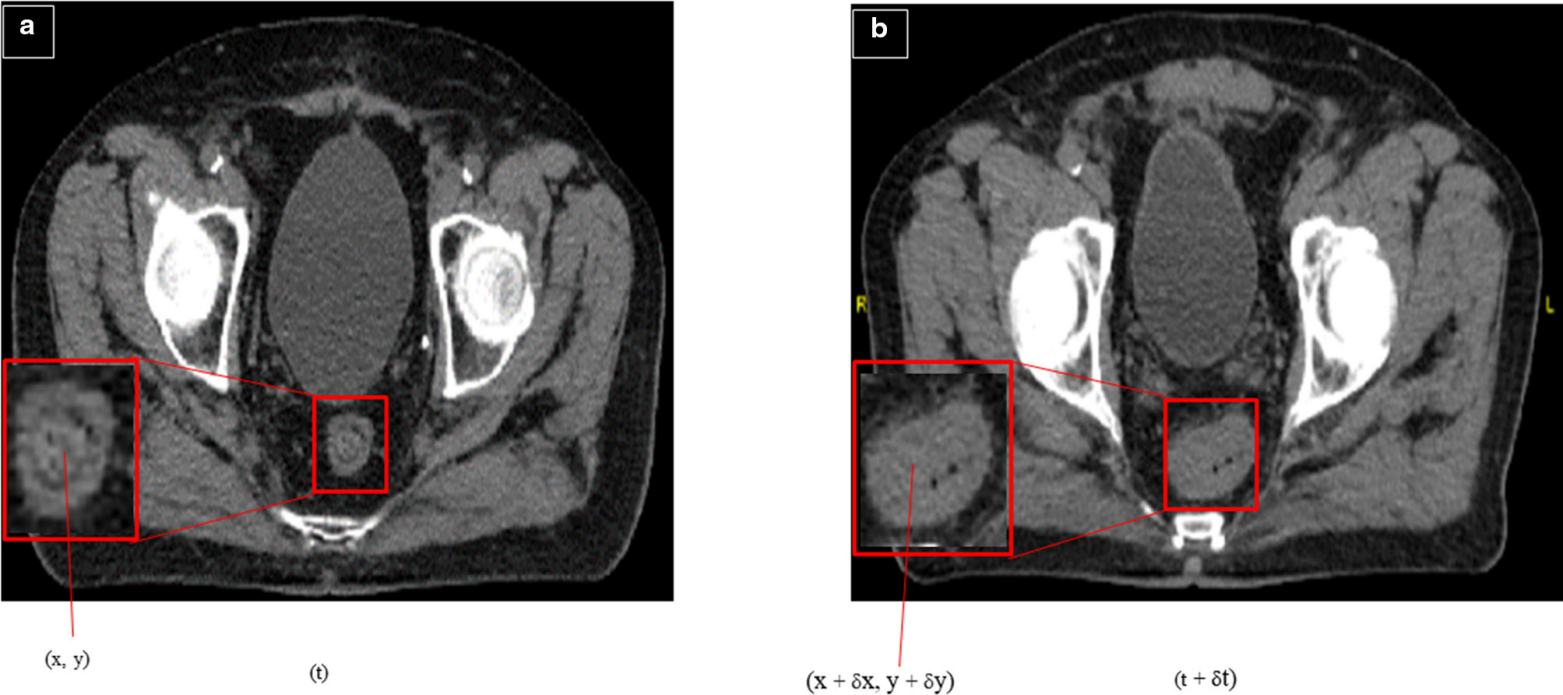
Zooming of rectum to extract a single point within each window. These windows are on two images of a pelvis. (**a**) is an image taken at time t and (**b**) is an image taken at time t plus ẟt when ẟt is small.

An optical flow is the apparent velocity distribution of brightness patterns in an image. An important information concerning the spatial organization of the objects observing the change rate of this arrangement can be obtained by the optical flow. It ascends from the relative motion of the observers and objects. ‘Figure 1’ demonstrates the concept of optical flow.^23^

As can be seen in the figure (8), the point in image (**a**) is (**x, y**), and at time (t+ ẟt), that point has moved to a new location, which is (x +ẟx), (y+ ẟy), (**b**). Thus, it is clear the displacement of the point (**x, y**) can be said to be (ẟx, ẟy). If $\left(\frac{\delta x}{\delta t},\frac{\delta y}{\delta t}\right)$ is taken, then it is essentially the speed of the point in the x and y directions is available. Therefore, (**u, v**) is called the optical flow corresponding to the point, which the optical flow algorithm tries to measure up.

Displacement (ẟx, ẟy), Optical flow: (u, v) = (ẟx/ẟt, ẟy/ẟt)

A couple of assumptions are made to solve the problem of the optical flow.

1. Image points remain constant in brightness over time. The brightness of objects in successive images remains constant as points move in space.
  I (x + ẟx, y + ẟy, t + ẟt) = I (x, y, t).
2. The spatial displacement (ẟx, ẟy) and the time step (ẟt) are assumed be small.^24^

###### Demons’ algorithm

Matching images using Demons algorithm relies on the theory of diffusing models. Two images are matched by treating one image as a deformable grid, the other images as a semipermeable membrane through which the deformable grid diffuses through the channels created by effectors inside these membranes. Demons' algorithm is an application of Maxwell’s Demons to clarify an inconsistency of thermodynamics developed in the late 19th century. Imagine a semi-permeable layer containing a set of 'demons' and separating two particles, a and b. Suppose also that this layer can distinguish between the various types of particles, and logically allow particle types a and b to diffuse to only their respective sides of the layer. Therefore, the only particles in A are a and b in B. In contradiction with thermodynamic principle number two, this results in a decrease in entropy. By distinguishing the particles, the demons generated an increased amount of entropy; therefore, the paradox solved and the total entropy system has increased.^25^

In theoretical form, a group of 'demons' operates on the voxels of the fixed image to cause the voxels in the moving image to be moved in conformity with the fixed image. Demons’ algorithm is used to calculate the vector field ((dr)= (dx, dy, dz)) to each pixel (r = (x, y, z)) that relates a moving image with a fixed image by using I_m_ (*r* + dr) = I_s_ (r)). There are six different versions of the demon’s algorithm, but they differ in how dr(x) is computed^9,26^

Where I*_m_* means the moving image intensity and I*_f_* means fixed image intensity.

###### Level-set algorithm

Level-set algorithm is another class of intensity-based method. Unlike optical flow, Curve/surface evolution theory is used to register two intensity images by evolving one image into another one by determining the velocity field of flow explicitly.^27^

In parametric form, if there are two images, which are source image I*_1_* (X) and target image I*_2_* (X). I*_1_* (X) is registered to I*_2_* (X) by evolving the level-sets that evolve along their normal till I_1_(X) has the characteristics of I_2_(X). Evolution is represented as:

Where S is the speed and ∇ is the gradient of the image. The evolution will stop when image I (X) changes from image I_1_(X) to image I_2_(X), so we must include a stopping mechanism in the rate term. Therefore, S = $I_{2}$ (X, t) - I(X). The vector field is calculated explicitly by the driving equation above. Therefore, velocity field ( ${\hat{V}}_{t}$ ) is

$$({\hat{V}}_{t})\frac{\left(I_{2}(X))-I(\vec{V}(X))I(\vec{V}(X)\right)}{||I(\vec{V}(X))||.\vec{V}(X,0)}=\vec{0}$$

### Point-based methods (spline algorithm)

Spline is a model that is used to classify spatial transformations. Mathematics defines a spline as a polynomial determined piecewise. Often used in computer graphics and computer-aided design, splines are popular because of the ease with which they can be constructed, the ease with which they can be evaluated, and their ability to approximate complex shapes through the designing of the interactive curve.^28^ Splines were originally used to model the surfaces of planes and ships using long strips of metal or wood by attaching different weights to them along their length to bend them. An example of using splines to represent spatial transformation is by applying two surfaces where vertical displacement links to the height above the plane.

For spline registration, matching landmarks or points must be identified in the target and source images. These points are called control points at which using splines, transformed images are mapped from the control point’s location in the target image into its counterpart in the source image by either approximating or interpolating displacements, which are represented as a whole a smoothly fluctuating displacement field. A spline-based mapping function is defined by either determining the control points of geometrical and structural landmarks identified in both images or using quasi-or pseudo landmarks (a regular mesh can be formed by placing control points equidistantly across an image).

The interpolation condition can be modeled as

where $x_{i}$ and $x_{i}^{`}$ symbolize positions of the control points in the target image and the source image, respectively (Hajnal & Hill, 2001).^29^

### Biomechanical-based modeling

Biomechanical-Based Modeling is a model that is used to model tissue distortions in image-guided radiotherapy. In this model, the finite element methods (FEM) is used to solve the partial differential equation (PDE) for elastic deformations. In this technique, the properties of rigid, fluid, and elastic structures are represented by a three-constituent model:

1. A triangular mesh with N knots is exploited to split the image for this purpose.
2. Anatomically-based labels are assigned to each knot based on their physical characteristics. Whilst rigid label is given to bone, CSF is given to fluid, but elastic label is given to soft tissues.
3. Rigid labels are Remained fixed, fluid and elastic labels are deformed by lessening a function of energy.

Deformations can be constrained by folding energy for fluid labels, but either a stiffness energy or a tension energy for elastic labels. This energy plays a central role in avoiding the transformation from the singularity’s development such as the folding or collapsing over of triangles.

The fold energy is calculated by:

The area of the undeformed triangle is represented by A_0_, the area of the deformed triangle is represented by A, and a threshold of energy is Υ .

The stiffness energy is calculated by:

The tension energy is calculated by:

$E_{tension}(\phi_{i},\phi_{j})={\left|\phi_{j}-\;\phi_{i}-\phi_{i,j}^{0}\right|}^{2}$ . $\phi_{i,j\;or\;k}$ represent nodes, $\phi^{0}i,j$ represents the relaxed space between two nodes.

A similarity measure is used to diminish the distance between matching points to devise the registration (Edward et al., 1998).

### Hybrid DIR

Hybrid methods include combining two DIR algorithms or using output of one algorithm such as deformable vector field (DVF) as the first condition to another one.^12,30^

### Calibration curve

There are two types of calibration curves used for dose calculation on kVCBCT, which are kVCBCT calibration curve and pCT calibration curve. kVCBCT calibration curves are derived through phantom/patient/population-specific measurements, while pCT calibration curves are derived from pCT images.

### Density override

kVCBCT images are overridden with either the CT densities or HU values from the CT images. Once kVCBCT HU values are overridden, the dose is computed on the modified kVCBCT images.^31^

### Combined techniques

Combining the kVCBCT DIR with HU override, or calibration techniques, the kVCBCT is modified.

### Deep learning algorithm

Deep learning is a subdivision of AI that belongs to the machine-learning branch. In deep learning, neural networks are used to teach the input data a specific task to yield hierarchical demonstrations of these data automatically. Deep learning has been proposed for image processing, especially generation of syntheses CT (sCT), which belong to convolutional neural networks (CNNs) class. Throughout training process, parameters learned by this process are used to combine convolutional filters, and multiple layers of filters are used to provide the depth. Uncovering parameters of the “optimal” model normalizes the training by the search criterion specified by a loss function ( L ).

**Figure 2.**
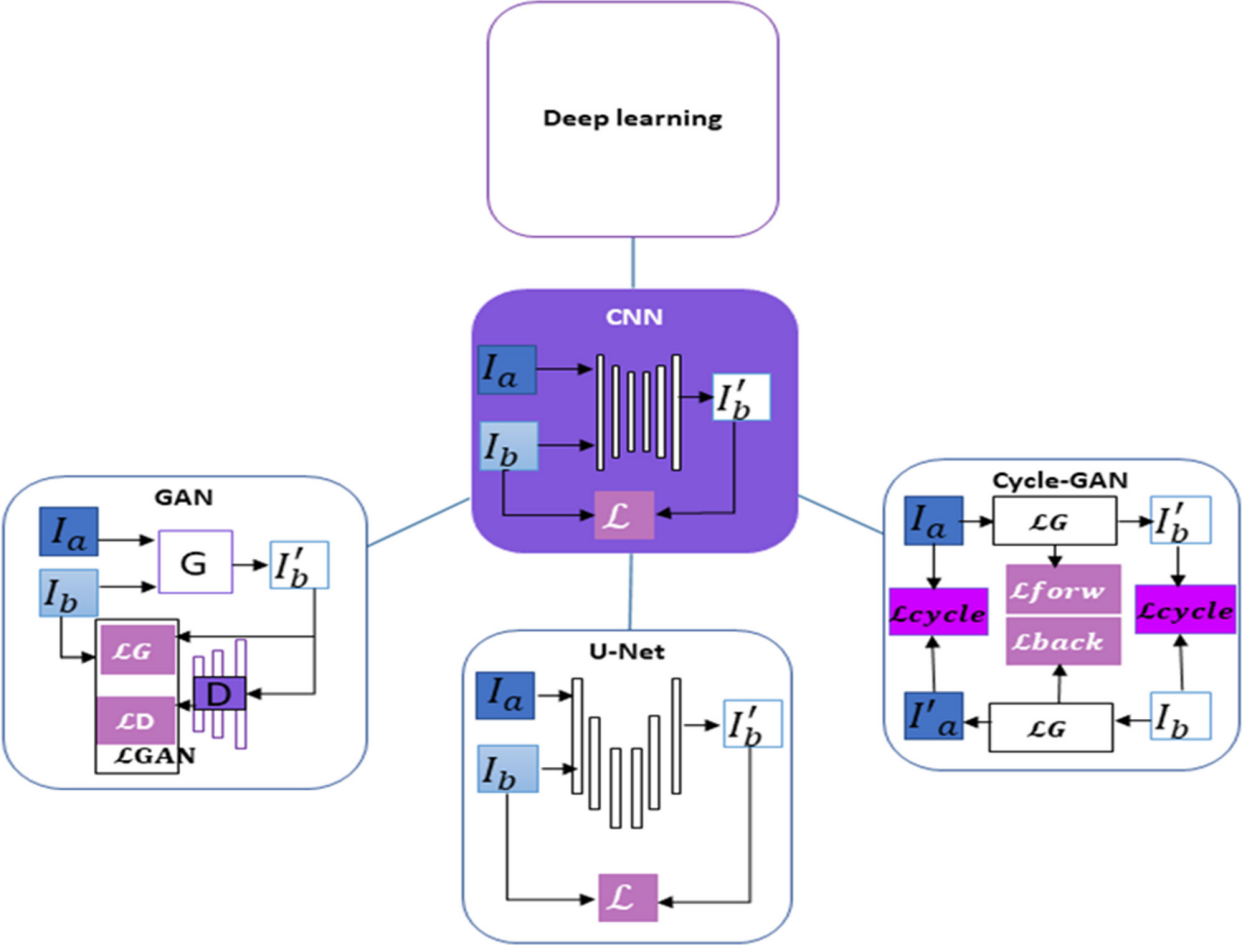
Demonstration of deep-learning technique used for translation of moving image to base image. Basically, a loss function between input data and output data is calculated in CNN and U-Net which are shown in the middle. Multiple loss and CNN in GANs are used to train the operation of generator which is shown in the left. Multiple cycle consistency losses and GANs in GANs are used to permit unsupervised learning which is shown in the right.

In deep learning, an image-to-image translation issue is used to devise medical image synthesis by uncovering a model that maps moving image (I_a_) to a base image (I_b_). The most widespread CNN-based architectures for medical image synthesis are generative adversarial networks (GANs), the U-nets and cycle-consistent GAN (cycle-GAN) 'Figure 2‘. GAN architecture uses two networks which are generator (G) and discriminator (D). D is trained to categories if synthetic images (I'_b_) generated by trained G are actual or false to enhance G’s performances. U-net architecture uses two paths, which are an encoding and a decoding path, in addition to skip connections. Paths and connections extract and restructure image characteristics, thus being taught to go from domain of I_a_ to I_b_. GANs are trained with a loss that compiles (GANs) and the U-nets resulting in true images. Complimentary advantages of these given bases can be taken into account by that many of GANs' parameters can be set, and U-nets is used as a generator in the framework of GAN. Cycle-GAN is a specific derivation of GAN in which unpaired image-to-image translation can be used. two GANs, which are forward pass and backward, are trained to yield two consistency losses L _C_ to diminish differences between I_a_ and 'I_b_, and I_b_ and I'_a_. In forward pass, GAN goes from moving I_a_ to I_b_, but in backward pass, GAN goes from I_b_ *to I_a_ to facilitate unpaired training*.^32^

## Methods

**Figure 3.**
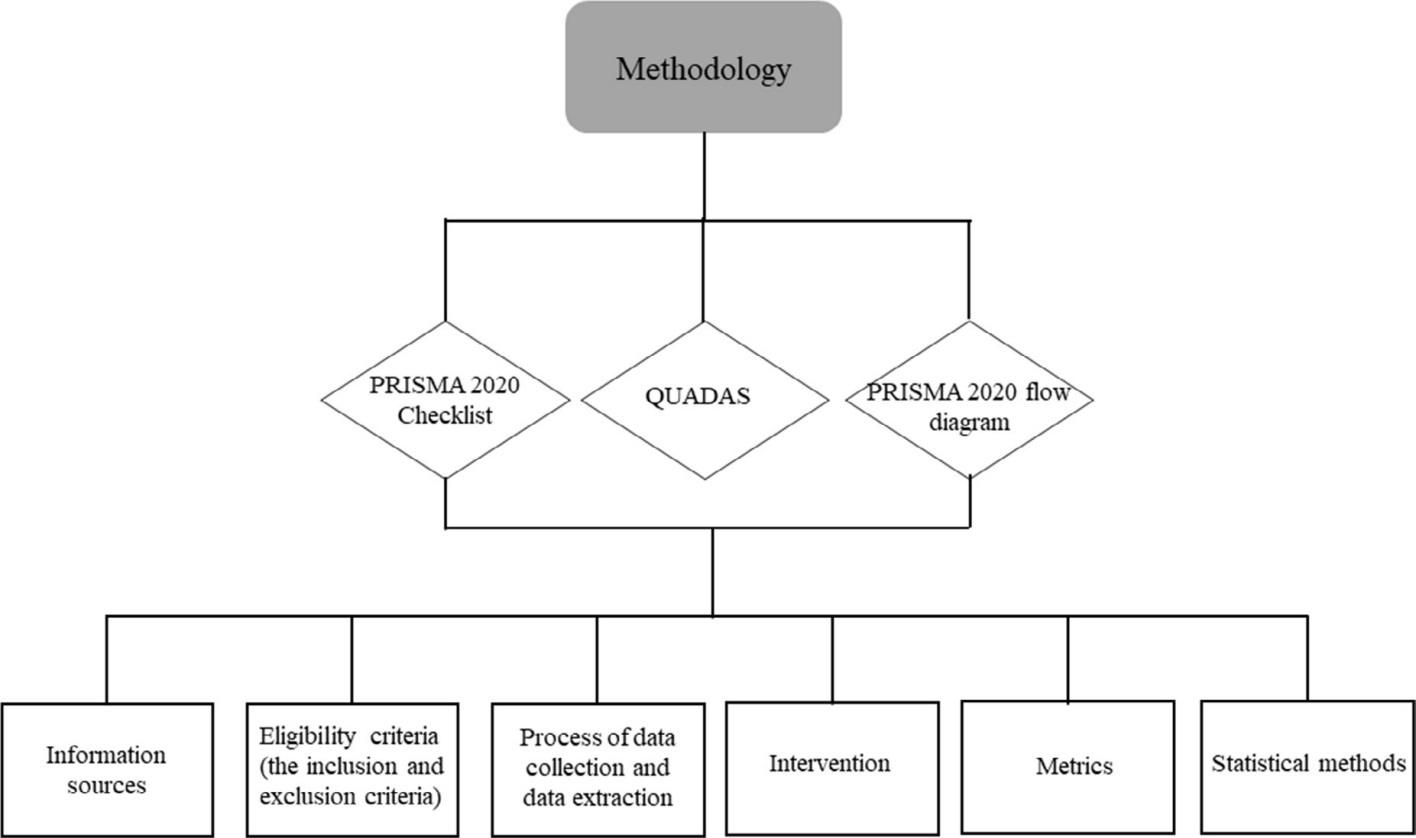
The design and methodology used.

The PRISMA revision was used in this study to process and report the data.^33^ The main documents adopted were ''^33^ Checklist'' and ''PRISMA 2020 flow diagram''. ‘Figure 3’ explains the design and methodology used in the systematic review. In this systematic review and meta-analysis, phantom studies and retrospective studies of cancer patients were incorporated using the following design: treatment is radiotherapy alone using linac equipped with kVCBCT. The studies that were included are case reports, retrospective studies, and experimental studies having consequences that influence the framework of radiotherapy.

### Information sources

Studies from 2005 onward that evaluated the kVCBCT feasibility for radiation treatment replanning in terms of dosimetric and geometric evaluation were located on the online databases (Google Scholar, PubMed, Scopus, EMBASE, and Science Direct). These systematic reviews are studies related to kVCBCT as tools for dose calculation and automated structures outlines. The search was taken in headings and a filter such as dosimetric evaluation. kVCBCT and contour propagation. The titles and abstracts of the included studies were not the only sources checked, but the bibliographies in the included studies were also examined. Both English and non-English language studies were searched from 2005 to 2022. 52 studies that evaluated kVCBCT images for radiation treatment replanning were included in the systematic review.

### Eligibility criteria (the inclusion and exclusion criteria)

From 2005 onwards, any trial evaluating methods of evaluating kVCBCT for dose calculation and geometric evaluation was included. Only comparative studies were reviewed in this field because no randomized controlled trials were available. The inclusion criteria used the following standards: (a) evaluations in calculating the dose using kVCBCT, (b) evaluating the geometric values of automated propagation of contouring structures on kVCBCT, (c) patients' data were analyzed and compared with standard data to calculate dosimetric and geometric metrices such as dose difference, γ analysis for dosimetric outcomes and Dice Similarity Coefficient (DSC) and registration error for geometric outcomes, (d) the study should be published in a journal with high impact factor, at least (3.5). The exclusion criteria used the following standards: (a) studies of graduate or postgraduate theses, animal experiments, and scholarly reviews, (b) repeatedly published literature or similar literature, (c) studies with good quality, but they have missing data, and (d) papers deals with dental kVCBCT. Authorship of studies was not blinded during the review process since it does not add any measure of quality. To ensure the validity of the results, the quality of the included studies was assessed according to the criteria suggested by Whiting et al.^34^ A Whiting assessment, which is called Quality Assessment of Diagnostic Accuracy Studies (QUADAS), consists of fourteen statements that have been framed as questions.

### Process of data collection and data extraction

Information was acquired by searching the databases specified above, and the abstracts or entire papers were evaluated by three reviewers (H.A.N, G.G, and N.B) independently. having read the title and abstract of potential eligible studies, articles were assessed and included based on the inclusion and exclusion criteria. After retrieving, reading and analyzing the articles, they were reviewed according to the guidelines.^33^ For instance, patients and results should accord with the review problem and object, respectively. Finally, study quality was determined according to criteria specified in (**section 2.2** study quality). Basically, the entire texts of articles were read for equivocal studies before making a decision. The results were then reviewed by a third investigator if there was still disagreement. A piloted form was used to extract valuable data from the studies included, with disagreements resolved through the exchange of ideas and discussion between study participants. Data extracted from each of the included studies are as follows:

(a) Study which includes first author last name and year of publication, (b) sample and sample size, (c) Journal name, (d) country, (e) software and algorithm, (d) application, and (e) metric value.

### Intervention

The kVCBCT images were examined for dosimetric and geometric evaluation. While Deformable Image Registration (DIR), deep learning algorithm, calibration curve, density override, intensity scaling, artefact corrected, hybrid method, and combined method were used for dosimetric evaluation, DIR and deep-learning algorithms were used for geometric evaluation. The performance of kVCBCT for dose calculation was compared to ground truth, which represents the fan CT with geometric features of kVCBCT. The performance of kVCBCT for automated contouring was compared with contour(s) drawn on kVCBCT by experienced physicians.

### Metrics

This evaluation is based on outcomes acquired from the plans prone to systematic errors. For dosimetric evaluation dose volume histogram (DVH) analysis, γ evaluation ( γ ) (2%, 2 mm) ≥ 90%, the dose differences (DD), dose similarity (DS) were used as a passing threshold. For geometric evaluation, DSC, the center of mass shift (CMS), the distance transform (DT), centroid position error (CPE), hausdorff distance (HD), distance to agreement (DTA), mean distance-to-agreement (MDA), the percentage error (PE), mean distance to conformity (MDC), normalized mutual information (NMI) and root mean squared error of the 3D canny edge (RMSEC), target registration error (TRE), the Jaccard index (JI), Error in flow endpoint (FEP), feature similarity index metric (FSIM),mean deformation difference (MDD in mm), mean mutual information difference (MID) mean absolute error (MAE), and the mean absolute differences (MAD) were used as a passing threshold.

### Statistical methods

Excel Package software was used for statistical analysis. Meta-analysis was conducted using a random-effects model to evaluate the accuracy of kVCBCT for treatment replanning. It included a mean 95% confidence interval (CI), weighted average, homogeneity test (Q) and *p*-value.^35^

The meta-analysis required studies to document the outcome of interest (*e.g.,* γ analysis and DSC score), along with a standard deviation (SD). Statistics were used to assess the 95% CI in cases where studies reported SD. Data from studies showing suitable outcomes were aggregated for meta-analysis. Therefore, the results of the statistical evaluation were denated as a mean with ±95% CI. Subgroup analyses were not performed since there were limited data on dose calculation and automatic region of interest (ROI) contouring using kVCBCT imaging.

The γ analysis score represents a vector metric of two components that merges differences in values of local dose (∆D) and DTA. A comparative study of matrices of calculated and reference dose distribution produces a matrix of *γ* values.^36^ In γ analysis*,* γ value of *≤*1:0 represents the pass rate. In this meta-analysis, γ value of ≥0.9 was considered a satisfactory value for adaptive radiation therapy.

The DSC score denotes an overlap index that is used to verify segmentation images and assess reproducibility. In DSC score, DSC of 0.0 means ‘'no overlap'' and DSC of 1.0 means ''complete overlap''. In this meta-analysis, a DSC score of ≥0.7 was considered a satisfactory value for adaptive radiation therapy.^37^

homogeneity test (Q) is ''a hypothesis test against the alternative that at least one effect size differs from the rest''.^35^ It is used to check the study if it can reasonably be expressed as distributing a normal effect size. In homogeneity test, Q of >75% refers that groups are heterogeneous. Q between 0 and 40% refers that groups are with low heterogeneity. If and only if a *P*-value is larger than 0.05, it means there is no significant presence of publication bias.

## Results

In this systematic review and subsequent meta-analysis, all methods designed to make kVCBCT feasible for dose calculation and contour propagation from CT into kVCBCT were assessed against gold standards. The gold standards are the fan CT with geometric features of kVCBCT and contour(s) drawn on kVCBCT by experienced physicians for the performance of kVCBCT for dose calculation and kVCBCT for automated contouring, respectively. The main focus of the meta-analysis was to produce an overall summary of the accuracy and efficacy of kVCBCT images for radiation treatment replanning. 1008 study abstracts were scrutinized for potential inclusion; 52 papers were recognized for further estimation. The articles were evaluated using the guidelines set out by Whiting et al.^34^ Overall, the included studies had satisfactory quality findings, except for patient spectrum, index scan review bias and uninterpretable scan results which scored 24%, 9%, and 24%, respectively, as shown in ‘Table 1’.

**Table 1.** Study quality grades for included articles

Number	Score	Statement	Yes	No	Not clear
**1**	Patient spectrum	Were the patients in the study representative of the ones who will be treated in practice?	0.24	0.59	0.20
**2**	Selection criteria	Criteria for selection were they spelled out?	0.83	0.11	0.07
**3**	Reference standard	Did the reference standard have a reasonable likelihood of correctly classifying the target condition?	0.76	0.11	0.13
**4**	Disease progression	Was the time between the index scan and the reference standard short enough for reasonable assurance that the target condition has not changed?	1.00	0.00	0.00
**5**	Work-up bias	Has the results of the whole sample or a randomly selected sample been verified against a reference standard?	0.85	0.04	0.11
**6**	Differential verification bias	Was the reference standard the same for all patients, regardless of their index scan results?	0.76	0.2	0.04
**7**	Independent reference standard	Did the index scan form part of the reference standard or was it independent of it?	0.98	0.00	0.02
**8**	Index scan execution details	Could the replicated process of the index scan be described sufficiently in detail?	0.63	0.24	0.15
**9**	Reference standard execution details	Could the replicated process of the reference standard be described sufficiently in detail?	0.57	0.33	0.11
**10**	Index scan review bias	Was the reference standard not known when index scan results were interpreted?	0.09	0.07	0.85
**11**	Reference standard review bias	Were the results of the reference standard analyzed without knowing what the index scan results were?	0.98	0.00	0.02
**12**	Uninterpretable scan results	In interpreting scan results, did clinicians have access to the same clinical data as they would have when the scan is used in clinical trials?	0.24	0.57	0.02
**13**	Study withdrawals	Results of uninterpretable / intermediate scans were reported?	0.98	0.02	0.00

**Figure 4.**
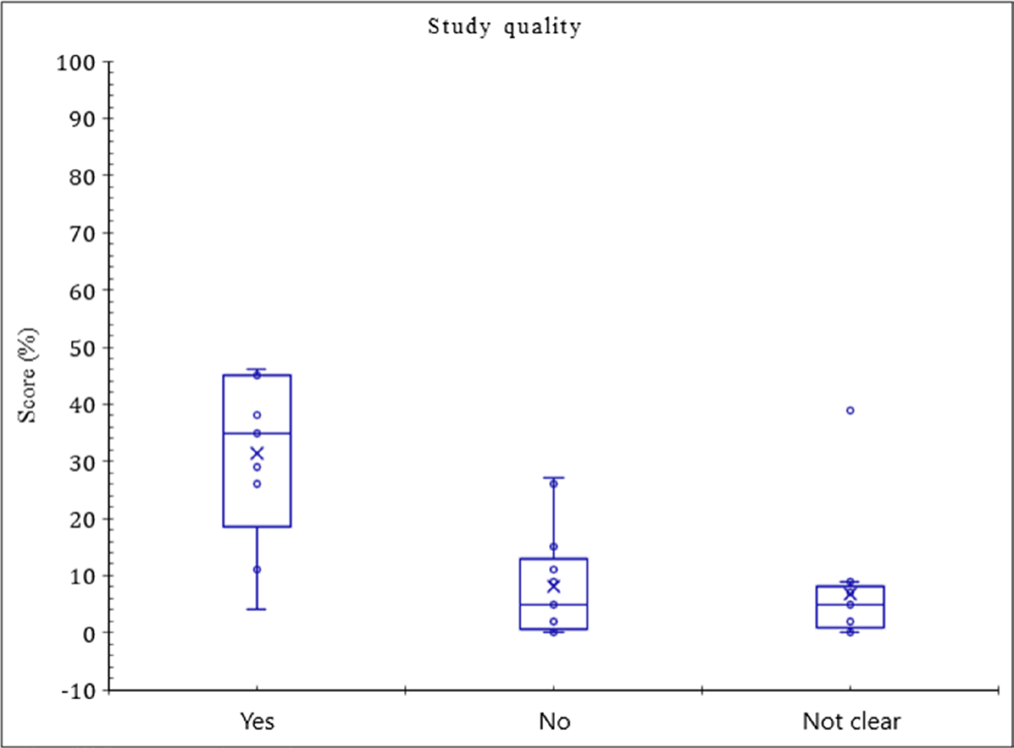
The methodological quality of included studies, as assessed by the QUADAS tool.

Figure 4 demonstrates box and whisker plot of QUADAS quality. Studies with a score of (yes) are more variable, especially at lower grades, and studies with a score of (not clear) vary much less than those with (No) or (yes). Therefore, it might seem that (not clear) coherence in relative grade would make predictions more dependable than the more variable (No) and (yes). Importantly, the score for (Yes) remained at the upper grade, which was about 50%, while for (No) and (Not clear) the score was 20 and 30%. There are also significant differences between the medians of (yes), (no) and (not clear) scores. Thus, about half of the grades in (yes) have a grade of 35%, (No) have 7 %, and (Not clear) have 9%. Most of the highest-scoring 25% in (yes) are at a higher level than the highest-scoring 25% in (no) and (not clear). As a result, (yes) beats both (not) and (not clear); thus, included studies fairly satisfied quality assessment.

All articles had low bias risks. Of the 13 domains, bias was likely to be present in the subdomains of index scan execution details (29 studies) and reference standard execution details (26 studies) because of not complete details of index scan execution and the use of composite reference standards.

### Literature search and study characteristics

After the computer and hand search using the strategy of search described, there were a total of 1008 studies. 800 studies were found by using computer search and 208 were found using hand search. Of total studies (1008), 863 were excluded after analyzing the title and abstract of each article because they were duplicated records, ineligible records, not in the field of interest, guidelines/reviews/books, case series/report or conference abstract, and they did not satisfy the inclusion and exclusion criteria. Therefore, 108 studies were sought for retrieval and nominated as included studies. Of these 108 studies, one study was further excluded because they were not retrieved and 38 studies were excluded due to insufficient data or other reasons. For instance, some studies did not verify the anatomical change based on the quantitative method while others did not use the standard reference for verification, but several studies used deformed CT that are not verified using a ground truth.

**Figure 5.**
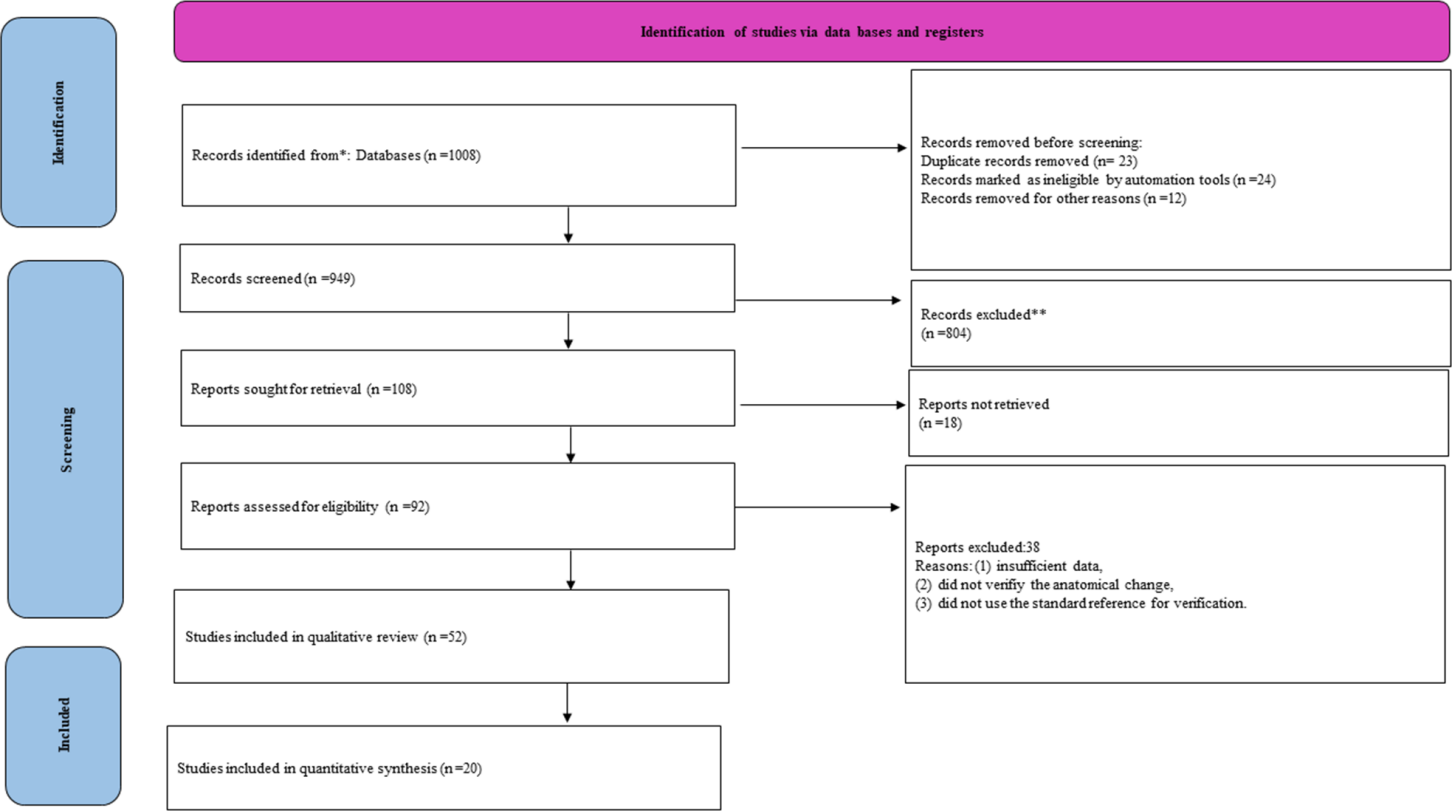
Diagram of the step-by-step process for the review and meta-analysis of the PRISMA database.

Finally, 52 studies were included. whilst 25 studies were included for the performance of kVCBCT for dose calculation, 27 were included for kVCBCT for automated contouring. 25 studies were conducted in European countries, 17 in the United State and 10 in Asia. Nine studies of dosimtric studies (using γ analysis as a metric) and 11 studies of geometric analysis (using DSC score as a metric) were qualified for inclusion for the meta-analysis since they come up with adequate quantitative data for the performance of the kVCBCT in radiation treatment replanning; for instance, studies have the γ values and DSC score accompanied by SD for the performance 'of kVCBCT-based treatment planning. ‘Figure 5’ depicts the systematic review and meta-analysis selected for this review and meta-analysis.

### The review of dose calculation methods based on kVCBCT

Studies that evaluated the accuracy and efficacy of CBCT for dose calculation commonly did so in terms of DVH and γ analysis for dose calculation. 25 studies were entitled for inclusion for quantitative synthesis because they were underlining the performance of kVCBCT for purposes of the dose calculation. The study characteristics and the demographics of the participants are in 'Table 2’. We reviewed the data from each study to establish metrics already described, but some publications did not have data, so re-analysis was difficult. An ideal technique would have a DVH metrics difference of 0% at a specific volume or point such as CTV, PTV, 95%, D98, and D2, and have 100% γ pass rate for dose. This level of accuracy is infeasible when kVCBCT images are used without correction. Therefore, many methods were developed to correct kVCBCT images. The types of methods used in these studies were categorized according to the model they used as follows: original kVCBCT,^38,55^ DIR,^10,16,17,39–41,43,46–48,52,56,59^ calibration methods,^9,10,42,44,50,53,57,58,60^ density override,^9,10,57^ intensity scaling,^45^ artefact correction,^51,56^ Artificial Intelligence (AI)^54^ and hybrid method.^41,59^ A clinically accepted tool would yield outcomes with difference of ≤ 2 and 90% GAMMA pass rate.^9^

**Table 2.** Included studies which used kVCBCT for dose calculation.

Study ID	Sample and sample size	Journal	Country	Software and algorithm	Metric	Value
(LEE et al., 2008)^38^	Phantom& H&N,1, 5	International Journal of Radiation Oncology* Biology* Physics.	The USA	Calibration curve	DVHs & dose distribution difference (ΔD) & dosimetric endpoints	ΔDmax was 0.5%, ΔD profile1%, but 3% at field edge.
(Eiland et al., 2014)^39^	H&N, 7	Journal of Radiation Research	Denmark	SmartAdapt, Demons algorithm	DVH analysis between CBCT& MCBCT	Mean Dmedian, D98%& D2% for GTV-T (5 Pts), GTV-N sin (4 Pts), GTV-N dxt (4 Pts), CTV-T (5 Pts), CTV-N sin (4 Pts) & CTV-N dxt (4 Pts), Parotid dxt (7) & Parotid sin (7) were 0.54% range (0–1.3%)
(Hay et al., 2020)^40^	H&N,20	(Medical engineering & physics)	The UK	Varian VelocityTM, B-Spline	Volumes Ipsilateral Parotid Gland Contralateral Parotid Gland, mean dose for the high risk planning target volumes (PTV_HR), Ipsilateral and contralateral, Max dose for PRV_Brainstem and PRV_Spinal Cord, D95% for the high risk planning target volumes (PTV_HR) and D95% < 95% for the high risk planning target volumes (PTV_HR) for resimulated CT and Deformed CT	Dose difference is about 0.023%, 1.7 and 1.5% between planning CT (pCT) and sCT for the high risk planning target volumes (PTV_HR), Ipsilateral and contralateral of Parotid Gland and Max dose of PRV_Brainstem and PRV_Spinal Cord, respectively.
(Irmak et al, 2020)^41^	H&N, 12	Zeitschrift für Medizinische Physik	Austria	ELASTIX (B-Spline), RayStation treatment planning system (TPS) (ANOCANDA), the population-based approach and Histogram Matching (HM) approach based on DIR (CBCTHM-D)& Histogram Matching (HM) approach on RR (CBCTHM-R)	γ analysis for deformed CT with ELASTIX (CTELX), ANOCANDA (CTANC), Histogram Matching based on DIR (CBCTHM-D), histogram matching based on rigid registration (CBCTHM-R)& population-based (CBCTPop) was used using the following criteria (10% of the prescribed dose is a dose threshold& γ criteria were applied: 2%/2 mm, 3%/2 mm,2%/3 mm and 3%/3 mm.	The results of γ criteria for CTELX, CTANC, CBCTHM-D, CBCTHM-R& CBCTPop using 2%/2 mm were 96.1 ± 3.9, 93.4 ± 6.3, 94.3 ± 5.7, 94.1 ± 5.0& 89 ± 8.3, using 3%/2 mm were 97.9 ± 2.4, 96.3 ± 4.0, 96.9 ± 3.9, 96.8 ± 3.3& 93.9 ± 5.7, using 2%/3 mm were 98.4 ± 1.4, 97.1 ± 3.0, 97.5 ± 2.0, 97.4 ± 2.1& 94.5 ± 5.4, using 3%/3 mm were 99.2 ± 0.8, 98.4 ± 1.8, 98.7 ± 1.2, 98.6 ± 1.2& 96.6 ± 3.6.
(MacFarlane et al., 2018)^42^	H&N, 15	AAPM	Canada	Patient-specific calibration (PSC) method, DIR (fast Demons) and density override	DVH& γ analysis with criteria (3%/3 mm) between corrected CBCT& rescan CT	The average dose differences for PSC, DIR& density override were 0.3%, 0.7–1.1%. The average γ pass rates with for PSC, DIR& density override were: 95.0, 96.1%& 94.4%.
(Otsuka et al., 2019)^43^	H&N, 10	*In Vivo*	Japan	Calibration curve between CT& CBCT	The dose difference between pCT and CBCT for parotid glands	The dose difference of ± 2.44% ±1.6 for the first fraction, ±1.44 ±3.6% for the second fraction and ranged from (1–10%) for the remaining fractions.
(Zijtveld et al., 2007)^44^	H&N, 2	Radiotherapy and Oncology	The Netherlands	HU mapping	γ analysis& dose difference	Gamma-values with 2%/2 mm& γ < 1 was 93–94.5% for patient1&2. The percentage of points with dose difference < 1%, dose difference < 2% were (84%–92% &81–90%) & (88%/96–87%/95%) for patient1&2, respectively.
(Kurz et al., 2015)^45^	H&N, 9	Acta oncologica	Germany	Morphons algorithm (vCT)& population-based CBCT intensity rescaling (CBCT LUT)	γ analysis using the following criteria g (3%, 3 mm)	For CBCT LUT γ analysis was (85–93%) and for vCT was 91–97%.
(Veiga et al., 2014)^46^	H&N, 5	AAPM	The UK	NiftyReg, B-Spline	The dose differences (DD) and γ analysis	The DD is smaller than 2% of the prescribed dose on 90% of the body’s voxels and it passes a 2% and 2 mm gamma-test on over 95% of the voxels.
(Disher et al., 2013)^47^	Phantom& Thorax,1, 2	DIR with HU override and substitution of CBCT pixel information with LED-sensitive CT.	Canada	DIR (ANIMAL) with HU override and DIR with substitution of CBCT pixel information with LED-sensitive CT.	Dose distribution difference (ΔD)	ΔD for HU override was (>= 9.5% Gy) & ΔD for substitution of CBCT pixel information with LED-sensitive CT was =< (−9.5%)
(Yuan et al., 2020)^48^	Thorax, 12	AAPM	The U.S.A	The Pinnacle TPS (research version 9.7), The Demons DIR algorithm	Isodose distribution comparisons Between virtual CT (vCT)‐based and re‐planning CT (rCT)‐based accumulated dose.	PTV Dmean, Lung‐CTV Dmean, Spinal cord Dmax, Esophagus Dmean& Heart Dmean are −0.03%, −1.10%, −5.55%, −0.80% and −1.46%.
(Cole et al., 2018)^49^	Thorax, 7	IOP (Physics in Medicine & Biology)	The UK	NiftyReg, B-Spline	DVH analysis& Voxel wise dose difference between deformed CT (dCT) & replan CT (rCT) (The region with prescribed dose > 95% & The region with prescribed dose > 50%<95%.	Mean percentage deviation of rCT structures for CTV + 1 cm max, CTV + 1 cm V95%, Spinal canal + 5 mm max, Lung max, Lung V20, Heart max & Heart V40 were 0.3%, 0.7%, 0.3%, 0.3%, 0.1%, 0.3–0.0%, max percentage deviations were 0.7%, 2.0%, 0.7%, 0.7%, 0.3%, 0.6–0.1%, mean percentage deviation of deformed structures for CTV + 1 cm max, CTV + 1 cm V95%, Spinal canal + 5 mm max, Lung max, Lung V20, Heart max & Heart V40 were 0.7%, 12.0%, 2.2%, 0.5%, 0.8%, 1.0–2.0% and max
(Kaplan et al,2018)^50^	Phantom + thorax, 1&12	Physics and imaging in radiation oncology	Denmark	Stoichio-metric calibrations	DVH analysis	For phantom study, mean dose and D2: for CTV-T were −0.8%& −0.8%, for CTV-N were 0% & −0.2%, for heart were −0.3%& −0.3%, for right lung were −0.8–0.1%, for bronchi were −0.7%&−2.7% and for spinal cord were 0.6–0.5%.
(Thing et al., 2017)^51^	Thorax, 21	Physics and Imaging in Radiation Oncology	Denmark	Artefact corrected in projection with DIR	DVH& γ analysis with criteria (1%/1 mm& 2%/2 mm) for clinical CBCT(cCBCT)& improved CBCT (iCBCT)	For cCBCT& iCBCT at 1%/1 mm level, γ pass rate was 79.9 %& 94.8%. For the cCBCT and iCBCT-based dose of all patients at 1%/1 mm, the mean of mean actual γ values was 0.79 ± 0.07 and 0.39 ± 0.03. For cCBCT& iCBCT at 2%/2 mm level, γ pass rate was 93.1–99.4%. For the cCBCT and iCBCT-based dose of all patients at 2%/2 mm, the mean of mean actual γ values was 0.39 ± 0.04 (mean ± standard error) and 0.21 ± 0.01 for the iCBCT-based doses.
(Almatani et al., 2016)^9^	Pelvis, 1	The British journal of radiology	The UK	Multilevel Threshold (MLT)	DVH& γ analysis with criteria (1, 3%/3 mm)	γ assessment for manual and automated MLT were 98.7 and 97.7%, respectively. 0.46% dose difference for manual MLT with 8 h operator time while the automated MLT algorithm showed –1.36%.
(Moteabbed et al., 2015)^52^	Pelvis Phantom,1	AAPM	The USA	PLASTIMATCH, B-Spline	DVH analysis and 3D γ analysis between pCT& mCBCT	The ∆D_mean_ averaged over (prostate, bladder, and rectum) for the bladder full scenario was 0.2%, The γ analysis showed 100% pass rate using 2%/2 mm and 1%/1 mm criteria for both warped CT to CBCT using calibration curve (wCT2-c), but 44.8–58.5% (1%/1 mm) pass for registering CT to CBCT without calibration curve (wCT2).
(Onozato, et al., 2014)^16^	Phantom& Pelvis, &10	International Journal of Radiation Oncology* Biology* Physics	Japan	AI version 2.7.0 software, B-spline algorithm	The difference of DVH between pCT and CBCT or deformed CT to CBCT modified with MLT (MCBCT_MLT_) or histogram matching (HM) (mCBCT_HM_) and γ evaluation	For the phantom, the differences in D_mean_ between PCT and CBCT or mCBCT regarding PTV, rectum, and bladder were 0.4%, 0.4%, or 0.1%, respectively. For the patient, the differences in D_mean_ between pCT& CBCT or MCBCT_MLT_ andmCBCT_HM_ is 3.0%, 2.5% or 0.9%. γ evaluation with a g value < 1 (1%/1 mm) was 97.0% in CBCT, 98.1% in CBCT with DIR, 99.0% in both mCBCT_MLT_ and mCBCT_HM_.
(Guan et al., 2009)^53^	Pelvis Phantom,1	IOP (Physics in Medicine & Biology)	The U.S.A	HU-electron density (ED) calibration	Difference of DVH, the central-axis dose (Dcax (cGy)), the mean dose (%), the maximum dose (%) & the minimum dose (%) for individual field(s), conformal and IMRT.	The maximum difference for fields AP, AP/PA, lateral fields were 1.8%, 2.3–6.7%, for conformal Dcax was 0.2%, Dmin, Dmax& Dmean for PTV were −5.1,–0.3& −0.3, for bladder were 2.2,–0.2& −1.1, for Rectum were −0.1,–0.5& −1.1.
(Sun et al., 2021)^54^	Pelvis, 120	Frontiers in Oncology	China	AI (CycleGAN), CycleGAN, UNET-GAN and FCN-GAN	DVH analysis	Difference of mean dose for PTV intestine, bladder, Femur-L& Femur-R are 3.7%, 3.7%, 3%, 3.7–3.3%, and D98%& D2% of PTV are3%& 4%.
(Giacometti et al., 2019) ^10^	H&N, thorax& pelvis,5,5 &5	The British journal of radiology	The UK	Standard pCT calibration curve, CBCT site-specific calibration curve, HU override& DIR (Modified Demons)	DVH analysis and 3D γ analysis between pCT& mCBCT	For pelvic node& prostate, metrics of target DVH were 0.6% for all techniques. For H&N patients, the largest differences for DIR, density override, site-specific calibration curve and the standard calibration curve are 2.7%, 3.2%, 3.9–5.4%, respectively. For lung, site-specific CBCT calibration& DIR, the difference is less than 1.9%. The γ analysis yields at least 80% pass-rates for minimum dose thresholds of 10%, 50%, 70% or 90%.
(Kaliyaperumal et al., 2017)^55^	Phantom, 1& H&*N* + Pelvis, 2	Journal of medical physics	India	Dose calculation on CBCT directly	DVH	For phantom, the dose difference between CBCT and CT plans of depth dose was in symmetric field& asymmetric fields within ± 1%. And ± 1.2%, but in the buildup region (for 15 MV < 2.5 cm& 6 MV < 1 cm). D95 for C‑shaped target was 95.7–94.5%. Maximum dose to normal structure was 61.6–59.5%. The mean dose was 32.1–31.7%. V95 for both was 99.8%. V5 was 108.5–109.2%. In patient cases, the dose difference for various points was less than 1%. For H&N D98 was 96.38–95.49%.
(Marchant et al., 2018) ^56^	Pelvis, H&N & thorax, 15, 14& 15	IOP (Physics in Medicine & Biology)	The UK	(Elastix& Niftyreg, B-spline) & Shading correction algorithm	Mean error and SD of PTV mean, PTV D95, rectum V40.8 and metrics combined using override ratio	For H&N, mean dose error and SD of PTV mean, PTV D95, rectum V40.8 and combined were less than 0.25% and less than 0.6% which are similar to Shading correction method. Both DIR algorithms led to few images with dose metric error greater than 2%. All dose metric errors in lung studies and pelvis are less than 2%.
(Yang et al., 2007)^17^	Phantom, Pelvis& thorax, 1,3 &1, respectively	IOP (Physics in Medicine & Biology)	The USA	Inhouse software, B-Spline	Percentage dose difference	For non-moved phantom the dose calculated using pCT agrees with that of CBCT-based calculation to within 1.0%. For moved phantom the discrepancy between the pCT- and CBCT-based calculations is about 3%. For prostate, the CBCT-reconstructed prostate dose agrees with the planned one to within 2%. For lung, the CBCT-reconstructed prostate dose agrees with the planned one to within 5%.
(Dunlop et al, 2015)^57^	H&N, 4, pelvis, 4& thorax, 3	Strahlentherapie und Onkologie	The UK	Calibration techniques (CBCT reconstruction (CBCT_r_)& density overrides where density assignment within a commercially available treatment planning system (RSauto),“water only” (W), “water and-bone” (WB)&“water-and-lung” (WL))	DVH& dose-difference maps	Dmedian, D95%, D98%& D2% of CTV in pelvic treatments (CBCTr was 0.8, 0.8, 0.8 & 1.2%), W was (0.3, 0.0,–0.1&0.7). WB was (−1.0,–1.5, −1.5&−0.7). Rsauto was −1.3,–1.5, −1.7&−0.8%. In H&N, CBCTr was 0.3, 0.5, 0.4,0.3& 0.7%. W was 0.4, 1.9, 0.4& 0.8%. RSaut was −0.1, 0.2,–0.1& −0.1%. In Lung, CBCTr was 2.4, 0.0& −1.3. W was −6.8,–7.0, −6.9& −6.2. WL was 0.4, 0.5, 0.3& 0.8. RSaut was −1.3,–1.1, −1.0& −0.9.
(Rong et al., 2010)^58^	Head, thorax& body phantom, 1	Medical Dosimetry	The U.S.A	HU-ED calibration	DVH, difference of mean dose, max dose& min dose	Difference of mean dose, max dose& min dose is 5.4%, 5.8–3.2% for body. 1.5%, 1.2–2.1% for head&N 0.5%, 1.0–0.1% for lung

The kVCBCT images were used to recalculate the dose for three groups of patients with cancers of head and neck (H&N), thorax, or pelvis. To calculate the dose for adaptive planning, nine studies focused specifically on the H&N while 11 studies focused specifically on the thorax, and nine studies focused specifically on the pelvis of 25 included studies. 25 retrospective applications of patients and ten experimental applications using phantom were conducted in these studies as displayed in ‘Table 2’ as well. 24 studies recalculated the dose on deformed CT or corrected kVCBCT and the ground truth was either resimulated CT on the same day of kVCBCT or planing CT if there are no significant geometric differences between CT and kVCBCT. The remaining study recalculated the dose on deformed kVCBCT and the ground truth was pCT.^39,47^

Two studies calculated the dose using original kVCBCT including two implementations of H&N and one study of the pelvis. Performance assessment of these studies in terms of the validated mean dose percentage difference, 95%, D98, and D2 and Δdmax was less than 2%, but it was 5% at field edge.^38,55^ 15 studies calculated the dose using kVCBCT corrected with DIR or DIR combined with another method including seven implementations of H&N, seven implementations of thorax, and five implementations of the pelvis. Performance assessment of these studies in terms of the validated dose percentage difference, 95%, D98, and D2 and Δdmax respectively ranged from 0.0 to 3%, 0.0 to 9.5%, and 0 to 2.7% for H&N, thorax and pelvis [.^10,16,17,39–41,46–48,52,56,59,60^ Eight studies calculated the dose using kVCBCT corrected with one of calibration methods including six studies of H&N, three studies of the thorax, and two studies of pelvis. performance assessment of these studies in terms of the validated dose percentage difference respectively ranged from 0.0 to 10%, 0.0 to 3.9% and 0.0 to 6.7% for H&N, thorax and pelvis.^10,27,42–44,50,53,57,58^ Two studies calculated the dose using kVCBCT corrected with density override including three studies of H&N, two studies of thorax and two studies of pelvis. Performance assessment of these studies in terms of the validated dose percentage difference respectively ranged from 0.0 to 3.2%, 0.0 to 7% and 0.0 to 1.5% for H&N, thorax and pelvis.^9,10,57^ One study calculated the dose using kVCBCT corrected with AI including one study of pelvis. Performance assessment of these studies in terms of the validated dose percentage difference respectively ranged from 0.0 to 3.7% for pelvis.^54^ The dose using kVCBCT corrected with artefact corrected, including one study of H&N, one study of thorax and one study of pelvis. Performance assessment of these studies in terms of the validated dose percentage difference respectively ranged from 0.0 to 2% for entire studies.^56^

Four studies calculated the dose using kVCBCT corrected with intensity scaling, artefact corrected and hybrid, but they documented rather than percentage dose difference. Therefore, they were also included for qualitative analysis as in ’Table 2 or analyzed using meta-analysis.^41,45,51,59^

**Figure 6.**
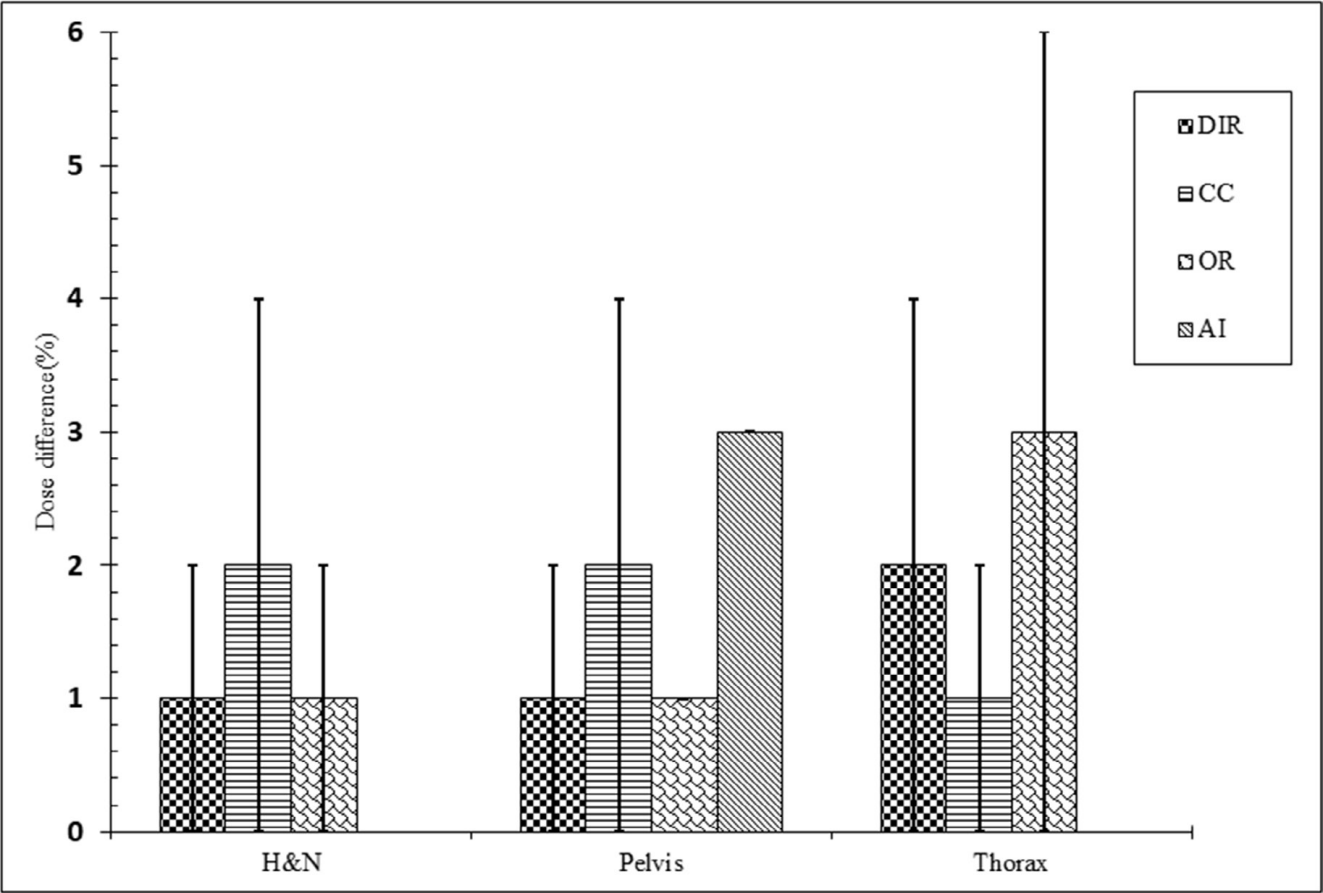
Mean dose differences for each correction method kVCBCT. The application of studies in three body regions is indicated. Legend: CC, calibration curve; OR override; AI, arteficial Intelligence.

Overall mean difference from the gold standard dose using kVCBCT depends on the type of technique. DIR and intensity override beat others techniques with mean dose difference and SD 1% and $\overset{-}{+}$ 1% for both H&N and pelvis, yet calibration curve-based method beat others with mean dose difference and SD 1% and $\overset{-}{+}$ 1% for thorax, respectively. In the case of calibration curve-based method, the mean difference and SD from the gold standard dose were 2% and $\overset{-}{+}$ 2% for H&N as well as pelvis, respectively. Also, mean differences and SD up to 2% and $\overset{-}{+}$ 2% in the dose for DIR was observed in thorax. Artificial intelligence and intensity override achieved the highest deference in dose. While Artificial intelligence had 3.1% $\overset{-}{+}$ 0 dose difference for pelvis, intensity override had 3.3 $\overset{-}{+}$ 3.1%. ‘Figure 6’ indicates the subgrouping of the studies based on the technique used.

#### Meta-analysis of the included studies

**Figure 7.**
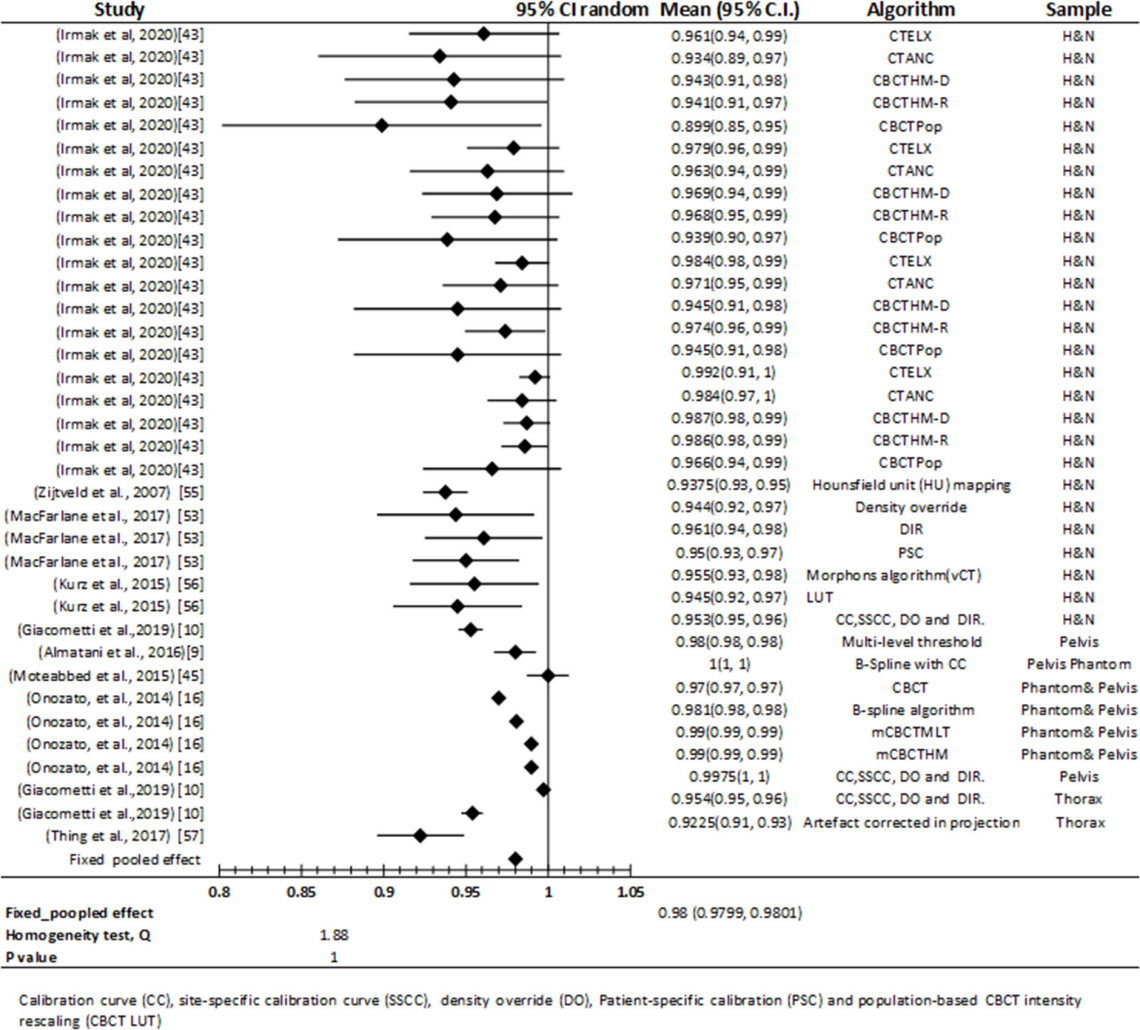
Forest plot of studies that assessed kVCBCT for dose calculation accuracy. For dose calculation, the results from the kVCBCT are centered around a γ pass rate of 0.98 with a 95% CI ranging from 97.799 to 98.01%. Legend: CI = confidence interval; calibration curve (CC); site-specific calibration curve (SSCC); density override (DO); patient-specific calibration (PSC) and populatio-based CBCT intensity (CBCTpop).

The collected meta-analysis comprised 36 applications of kVCBCT for dose calculation, expressed in nine individual studies of included studies. Evaluation of the studies included in Meta-analysis performed in terms of γ analysis. Hence, meta-analysis revealed an overall γ pass rates of 0.98 (95% CI:0.9799–0.9801), and Heterogeneity test (Q) have 1.88 Q-value and 1 *P*-value, indicating that there is no significant inhomogeneity ‘Figure 7’.

For individual studies of dose calculation based on kVCBCT, the outcomes are also depicted in ‘Figure 7’. Performance estimation of these studies in terms of the validated γ pass rates respectively ranged from 89.9% (95% CI:85–95%) to 99.92% (95% CI:91–100%) for H&N, 92.25% (95% CI:91–93%) to 95.4% (95% CI:95–96%) for thorax and 97% (95% CI:97–97%) to 100 (95% CI:100–100%) for pelvis.

### The review of the segmentation of organs in cone beam CT

Studies that evaluated the accuracy and efficacy of kVCBCT for contour propagation did so in terms of DSC, CMS, DT, CoM, HD, RAVD, MDA, PE, and CPE for contour propagation. 27 studies were included because they were underlining the performance of kVCBCT for purposes of automated contouring ‘Table 3’. ‘Table 3’ provides study characteristics and participant demographics based on analysis of the full-texts of these 27 included studies in the systematic review. Four groups of patients with cancer of the H&N, thorax, pelvis, or abdomen were recontoured using kVCBCT images. To automatically recontour the structures for treatment replanning, 15 studies focused on the H&N while 12 studies focused on the thorax, and seven studies focused specifically on the pelvis, but two studies dealt with the abdomen. 25 retrospective studies of patients and two experimental studies using phantom were expressed in 27 included studies as displayed in ‘Table 3’ **as** well. 26 studies propagated the contour from CT to kVCBCT and the ground truth was contour drawn on CT by expert. The one study propagated the contour from kVCBCT to CT and the ground truth was contour drawn on CT by expert.^75^ Two techniques were used to contour structures on kVCBCT, including DIR and AI. While the former was used to contour the structures for twenty-two studies in which 13 studies involved H&N, ten studies involved pelvis, six studies involved thorax and two studies involved abdomen, the latter was used to contour the structures for five studies in which three studies involved H&N and two studies involved pelvis. An ideal tool would produce results as good as in the case of registering the same images of the same modality (CT or kVCBCT). Hence, the average registration error between manually contoured ROI-surfaces and automatically contoured ROI-surfaces should be 2.0 mm±5 mm, 2 mm and 3 mm in head-and-neck, the thorax and pelvis cancer cases, respectively, and DSC of (≥ 70%).^35,80,81^ Most of registration errors were estimated based on the difference between calculated motion field and known motion field, manually drawn contour and manually drawn contour, center of masses, the distances to the closest point, or edges of deformed images and reference images. CMS, DT, CoM, HD, RAVD, MDA, PE, CPE and RMSEC were considered as equavalent quantity to each another, and lower values of them indicate a better agreement between the corresponding images.

**Table 3.** Included studies that uses kVCBCT for contour propagation

Study ID	Sample and sample size	Journal	Country	Software and algorithm	Metric	Value
(Liang at al., 2021)^61^	H&N,124	arXiv preprint arXiv	The U.S.A	CycleGAN (Joint model)	DSC, relative absolute volume difference (RAVD), and 95% HD	Average DSC was 0.83 ± 0.09, average RAVD was 0.108 mm and average HD95 was 2.01 ± 1.81 mm.
(Eiland et al., 2014)^39^	H&N, 7	Journal of Radiation Research	Denmark	SmartAdapt, Demons algorithm	DSC and CMS	DSC& CMC for GTV-T (5 Pts), CTV-T (5 Pts), GTV-N dxt (4Pts), CTV-N dxt (4 Pts), GTV-N sin (4Pts), CTV-N sin (4 Pts), Parotid dxt (7 Pts), Parotid sin (7 Pts) and Spinal cord (7 Pts) were 0.75 ranged (0.44–0.86) & 3.6 mm ranged (1.9–7) mm, respectively.
García-Mollá et al., 2015) 59	H&N, 5	Physica medica	Spain	Raystation TPS, ANOCANDA	The distances between six hundred (600) points of interest (POIs)	The POIs in rigid and soft areas had an average distance of 1.4 ± 0.5 mm and 2.0 ± 0.1 mm, respectively.
(Veiga et al., 2014) ^46^	H&N, 5	AAPM	The UK	NiftyReg, B-Spline	DSC, DT and CPE.	A mean value of 0.850 in DSC was achieved in overlap, and mean DT was 0.8 ± 0.3 mm over all structures. A mean value < 2 mm in CPE was achieved.
(Veiga et al., 2015)^62^	H&N, 5	AAPM, medical physics	The UK	NiftyReg, B-spline with different properties standard asymmetric registration in the forward direction followed by the numerical estimation of the inverse of this transformation (DIRsas + inv), (Standard asymmetric registration (DIRsas + sas), Symmetric registration parameterized by a stationary velocity field which inherently (DIRsvf)), Inverse-consistent symmetric registration (DIRics).	DSC, false	Overall DSC for DIRsas + sas, DIRsas + inv, DIRics& DIRsvf were 0.851 ± 0.080, 0.847 ± 0.082, 0.848 ± 0.075& 0.851 ± 0.073. FP for DIRsas + sas, DIRsas + inv, DIRics& DIRsvf were 0.17 ± 0.11 mm, 0.15 ± 0.11 mm, 0.18 ± 0.12 mm& 0.15 ± 0.10 mm. FN for DIRsas + sas, DIRsas + inv, DIRics& DIRsvf were 0.14 ± 0.09, 0.15 ± 0.10, 0.14 ± 0.08& 0.15 ± 0.09. DT2mm (%) were 9 ± 6 mm, 10 ± 6 mm, 10 ± 6 mm& 9 ± 6 mm. DTmean (mm) were 0.3 ± 0.4 mm, 0.4 ± 0.4 mm, 0.2 ± 0.4 mm& 0.3 ± 0.4 mm. DTstd (mm) were 1.3 ± 0.4 mm, 1.3 ± 0.3 mm, 1.3 ± 0.4 mm, 1.3 ± 0.4 mm. DT95% (mm) were 2.7 ± 0.9 mm, 2.7 ± 0.9 mm, 2.8 ± 0.9 mm& 2.7 ± 1.0 mm.
(Chen et al., 2021)^63^	H&N, 40	Frontiers in AI	China	Deep learning algorithms, a	DSC, HD, and center of mass (COM) displacement of original CBCT (oCBCT)-to-(rCT) and enhanced CBCT (eCBCT)-to-rCT)	Mean DSC of oCBCT-to-rCT was (0.70 ± 0.13), eCBCT-to-rCT was (0.83 ± 0.06). Mean HD of oCBCT-to-rCT was (7.2 ± 2.5 mm), eCBCT-to-rCT was (4.2 ± 1.3 mm). Mean COM of oCBCT-to-rCT was (4.4 ± 2.2 mm) & eCBCT-to-rCT was (2.8 ± 1.9 mm).
(Bendall etal., 2015) ^64^	H&N, 20	Acta Oncologica	The UK	Velocity AITM (Version 3.1.0, Varian Medical Systems) and MIMTM Maestro (Version 6.4.3, MIM Software Inc., Cleveland, OH, USA) (MIM)	The volume ratio (VR), maximum, mean and SD of HD, DTA and normalized DSC (nDSC).	VR of OAR was 0.6–1.4. HD in contouring of RO ranged between 3.0 and 17.4 mm. The mean of ST of DTA was 1.6 mm. For MIM& velocity, individual OARs with nDSC >= 1 was 29–19%, respectively.
(Hou et sal., 2011) ^65^	H&N, 12	AAPM, medical physics	The U.S.A	The Insight Toolkit (ITK), Demons	TRE& the volume overlap index VOI between synthesized ROI contours& manually delineated ROI contours.	TRE of soft tissue and bony structures were s 2.8 0.2 mm& 2.4 0.2 mm, respectively. TRE for over all points of patient cases and anatomy was 2.60.6 mm. The average VOI was 76.2 4.6%
(Rosen et al., 2018)^66^	H&N, 128	International Journal of Radiation Oncology* Biology* Physics	The USA	SmartAdapt, Varian Medical Systems, Palo Alto, CA, Demons algorithm	DSC, MDA, and SD of the MDA (MDA SD)	DSC (s) of parotid glands was 0.83, MDA were 1.7 mm, and MDA SD was 1.8 mm.
(Li et al., 2016)^67^	H&N, 6	Technology and Health Care	China	DIRART (Optical flow, Demons, Level-set and Spline)	DSC	Mean, median DSC for OARs of the combined Horn-Schunck and Lucas-Kanade (HSLK), the Original Horn and Schunck (HSO), the Horn and Schunck with Issam’s non-linear smoothness (HS_INLS), the original level set motion (OLSM), the free form deformation (FFD), the double force demons (DFD) and the iterative optical flow (IOF) were (0.765 ± 0.121, 0.777), (0.758 ± 0.121, 0.780), (0.758 ± 0.121, 0.780), (0.758 ± 0.122, 0.790), (0.755 ± 0.117, 0.756), (0.705 ± 0.119, 0.717)& (0.753 ± 0.115, 0.773).
(Kearney et al., 2018)^68^	H&N	Physics in Medicine & Biology	The U.S.A	Convolutional neural network-based algorithm, deep convolutional inverse graphics network (DCIGNs)	FSIM, NMI & RMSEC	Mean FSIM was 0.92, mean NMI was 0.653& RMSEC was 0.175 mm for DCIGNs
(Zhong et al.,2015)^69^	Thorax, 3	In World Congress on Medical Physics and Biomedical Engineering	The U.S.A	VelocityAI (v3.0.1, Varian Medial Systems, Palo Alto, CA), B-spline + a finite element method (FEM)	The difference between these points on deformed and fixed image	The average difference for hybrid was 1.5 mm.
(Samavati et al., 2016)^70^	Thorax, 10	AAPM, medical physics	Canada	MORFEUS (biomechanical model)& Hybrid (biomechanical model + B-Spline)	DSC& TRE	DSC of MORFEUS& Hybrid for tumor are 0.73& 0.86, respectively. TRE of MORFEUS& Hybrid for tumor are 2.8 ± 1.6 mm& 1.2 ± 1.0 mm.
(Cole et al., 2018)^49^	Thorax, 7	IOP, PMB	The UK	NiftyReg, B-Spline	DSC, CPE and DT	DSC for scapulae, spinal cord& trachea were 0.883 ± 0.011, 0.898 ± 0.014, 0.88 ± 0.03, 0.889 ± 0.021. CPE for scapulae, spinal cord& trachea were 3.0 ± 1.4 mm, 1.6 ± 1.0 mm, 1.4 ± 0.7 mm & 2.0 ± 1.3 mm. |DT| 95% for scapulae, spinal cord& trachea were 1.8 ± 0.3 mm, 1.8 ± 0.4 mm, 3.2 ± 1.0 mm & 2.2 ± 0.9 mm. |DT| mean for scapulae, spinal cord& trachea were 0.59 ± 0.07 mm, 0.61 ± 0.07 mm, 1.1 ± 0.4 mm & 0.7 ± 0.3 mm. |DT| s.d. were 0.73 ± 0.12 mm, 0.64 ± 0.08 mm, 1.3 ± 0.6 mm & 0.8 ± 0.4 mm.
(Foley et al., 2016)^71^	Pelvis, 3	Physica Medica	The UK	Scilab 5.5.0, a 3D Phase correlation algorithm in Fourier domain	DSC& the mean error and SD	DSC of rectum arranged from 0.05 to 0.46, & for bladder arranged from 0.04 to 0.09. The average value of the mean 3D error (±σ) was 2.35 (±1.54) mm.
(Anaya et al., 2019)^72^	Pelvis, 18			SmartAdapt DIR, Demons algorithm.	DSC, CMS and MDC.	The DSC and CMS for prostate only were 0.81 ± 0.06 and 3.5 ± 2.0 mm, but DSC and CMS (cm) for rectum were 0.69 ± 0.08 and 8.1 ± 4.6 mm, respectively. For rectum and CTV, CMS were 1.54 ± 0.76 and 2.68 ± 1.17, respectively.
(Imae et al., 2020) ^18^	Pelvis, 20	Radiological Physics and Technology	Japan	Raystation TPS, ANOCANDA	DSC for Prostate, proximal seminal vesicle, Bladder Rectum, Left femoral head and right femoral head	DCSs in post-DIR were greater than 0.8 for all patients.
(Brion et al., 2021)^19^	Pelvis, 134	Computers in Biology and Medicine	Belgium	Source only, adversarial networks (3D U-Net) and intensity-based data augmentation	DSC	DSCs of Adversarial networks was 0.660, 0.447& 0.787 for prostate, rectum, &the bladder. Source only was 0.629, 0.179 and 0.749. brightness-based data augmentation was 0.734, 0.701 and 0.837.
(Xie et al., 2008)^73^	Pelvis, 2	AAPM	The USA	The Visualization Toolkit (VTK), thin plate algorithm	DSC	For rectum, DSC was 0.9.
(Hart et al., 2017)^74^	Pelvis, 10	Physics in Medicine & Biology	The USA	Demons with optimizing variable-kernel smoothing parameters	DSC	DSC for bladder, prostate& rectum were 98%, 97–96%
(Takayama et al., 2017)^75^	Pelvis, 10	Journal of Radiation Research	Japan	Raystation TPS, ANOCANDA& Intensity-based algorithm	DSC& center of mass (COM)	The average DSCs for intensity-based DIR for the prostate, rectum, bladder, and seminal vesicles were 0.84 ± 0.05, 0.75 ± 0.05, 0.69 ± 0.07 and 0.65 ± 0.11, respectively, and for hybrid DIR were 0.98 ± 0.00, 0.97 ± 0.01, 0.98 ± 0.00 and 0.94 ± 0.03, respectively. Average COM displacements for intensity-based DIR for the prostate, rectum, bladder, and seminal vesicles were 3.1 ± 1.5 mm, 4.1 ± 1.4 mm, 7.9 ± 2.2 mm and 3.6 ± 1.2 mm, whereas those values for hybrid DIR were 0.1 ± 0.0 mm, 0.3 ± 0.2 mm, 0.2 ± 0.1 mm and 0.6 ± 0.6 mm, respectively (*p* < 0.05).
(Weistrand et al., 2015)^12^	H&N, Thorax& pelvis, 1, 16& 2	AAPM	Sweden	Raystation TPS, ANOCANDA	DSC& image similarity	For lung, mean DSC was 0.98& 0.99 for Lt& Rt lungs. For pelvis DSC is 0.97 for bladder, 0.97 for prostate, 0.94 for rectum. For H&N is 0.8 for Lt parotid, 0.85 for Rt parotid. For lung, rostate and H&N, mean image similarity was 0.95, 0.83 and 0.76, respectively.
(Woerner et al., 2017)^20^	H&N,6, pelvis,5& Abdomen, 5	Technology in cancer research & treatment	The USA	Velocity Advanced Imaging 2.8.1 (Varian Medical Systems, Palo Alto, California), B-Spline	DSC, mean surface distance (MSD), and HD	Overall mean DSC for all organs 0.79. Mean DSC for H&N, prostate, and pancreas groups were 0.77, 0.74, and 0.84, respectively. Mean agreement 2.24 mm for DIR-to-physician contours. HD was 12.0 mm.
(Léger et al., 2020)^43^	Pelvis, 74 for CT& 63 CBCT	Applied Sciences	Belgium	The 3D U-net fully convolutional neural network	DSC and JI	DSC of prostate, rectum& bladder were 0.758 ± 0.101, 0.814 ± 0.055&0.787 ± 0.131. JI were2.47 ± 1.93, 0.690 ± 0.077&2.47 ± 1.93.
(Zachiu et al., 2017)^76^	Abdomen 1& thorax, 1	Physics in Medicine & Biology	The Netherlands	Evolution registration algorithm,	FEP	For kidney registrations, the FEP is 1.18 ± 0.3 mm, and for lung registrations is 0.94 ± 0.3 mm.
(Li et al., 2011)^77^	Pelvis, 10, lung, 5, head& neck, 5,thorax, 10.	AAPM, APPLIED CLINICAL MEDICAL PHYSICS.	China	The Insight Toolkit (ITK), normal B-spline (NFFD) & normal B-spline with edge-preserving scale space(EFFD).	MDD (mm), MID with ground truth (deformation1 (D1), deformation 2 (D2)& deformation 3(D3)& DSC.	MDD for H&N, chest, lung, breast, prostate& rectum were 0.473 mm, 0.590 mm, 0.658 mm, 0.593 mm, 0.539 mm& 0.548 mm for D1. 0.496 mm, 0.646 mm, 0.660 mm, 0.613 mm, 0.552 mm& 0.550 mm for (D2). 0.514 mm, 0.648 mm, 0.664 mm, 0.617 mm, 0.570 mm& 0.568m for D3. MID for H&N, chest, lung, breast, prostate& rectum were 0.903, 0.731, 0.694, 0.742, 0.823& 0.833 for (D1). 0.896, 0.704, 0.675, 0.716, 0.819& 0.814 for (D2). 0.867, 0.727, 0.677, 0.701, 0.818& 0.821 for (D3). DSC is 95%.
(Chao et al., 2008)^78^	Rectum, 5& digital phantom, 1.	Physics in Medicine & Biology	The U.S.A	The Insight Toolkit (ITK)& the Visualization Toolkit (VTK)	Comparison synthetic contour on phantom with synthetic contour on the patient	The difference between two sets were 1.3 mm & 2 mm, respectively.
(Yu et al., 2015)^79^	Phantom, 1, H&N, 3& thorax, 3	Physics in medicine & Biology	China	OpenCL parallel programming, (the Demons algorithm and gradient-based free form deformation algorithm (GFFD))	MAD and SD, MAE and DSC	The MAD and SD of grid size maximum deviations (β = 4) were 1.54 ± 1.59 mm, 1.36 ± 1.59 mm& 0.77 ± 1.32 mm for non-registration, demons& GFFD registration. The MAD and SD of (β = 6) were 2.03 ± 1.94, 1.43 ± 1.85& 0.87 ± 1.48. The MAD and SD of (β = 8) were 2.70 ± 2.28, 1.56 ± 2.01& 1.05 ± 1.62. MAE edge of (β = 4) were 1.37, 0.48 & 0.16 for non-registration, demons& GFFD registration. The MAD and SD of (β = 6) were 1.38, 0.5& 0.17. The MAD and SD of (β = 8) were 1.59, 0.48 & 0.18. The MAD and SD of head were 2.3 mm, 1.35 mm & 1 mm for before registration, Demons& GFFD. The MAD and SD of lung were 3.4 mm, 1.9 mm& 1.33 mm for non-registration, Demons& GFFD. DSC for Demons was 90% for GTV, and 94–96% for GTV & parotid gland.

Twenty-two studies used for automatic region-of-interest delineation using DIR including 12 studies of H&N, seven studies of thorax, ten studies of pelvis and two studies of abdomen. The included studies compared automatic region-of-interest contour with manual region-of-interest contour for the treatment target and the critical organs at risk. For instance, this ROI includes tumor, parotid glands and spinal cord in H&N cancer cases, vertebrae, external contour and sternocleidomastoid muscles, and tumor, bladder and rectum in pelvis cancer cases. Performance assessment of these studies in terms of the registration error ranged from 0.59 ± 0.07 to 3 ± 1.4 mm, 0.1 ± 0 to 7.9 ± 2.2 to 0.1 and 1.18 ± 0.3 to 12 mm for H&N, thorax, pelvis and abdomen, respectively. Furthermore, five studies used for automatic region-of-interest delineation using AI including three studies of H&N, two studies pelvises. According to the performance assessment of these studies, the registration errors ranged from 0.175 to 4.2 ± 1.3 mm for H&N. Seven studies were used for automatic region-of-interest delineation using DIR or AI, but they documented rather than registration error. Therefore, they were also recorded for qualitative analysis as in ’Table 3 or analyzed using meta-analysis.^12,13,62,73,82,83^

**Figure 8.**
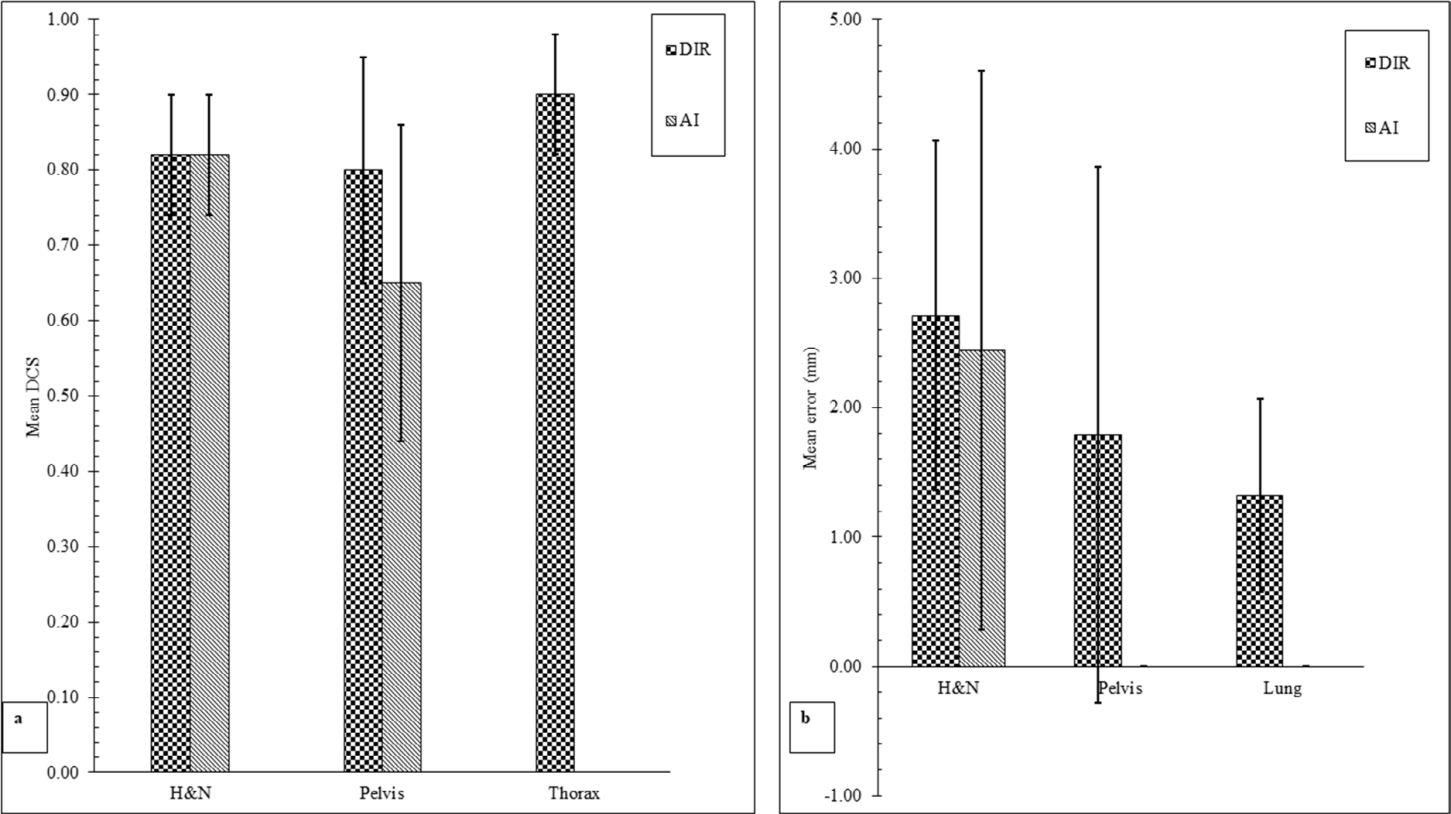
Capacity of different methods for recontouring of ROI on kVCBCT. (**a**) Mean DSC of DIR and AI for three regions, including H&N, pelvis and thorax. (**b**) Mean error of DIR and AI for three regions, including H&N, pelvis and thorax.

'Figure 8’ indicates the subgrouping of the studies based on the technique used. Overall mean difference from the gold standard of DSC using kVCBCT depends on the type of technique. DIR and AL scored the same DSC of 0.82 ± 80.0 for H&N, yet DIR beat AI with mean DSC score ± SD of 0.8 ± 1.5 for pelvis. DSC ± SD of AI was on the other hand 0.65 ± 0.21 for pelvis. No study of AI was included for thorax. Not only DSC was used to evaluate accuracy of automatic ROI outlining, but registration error was also used in the included study as a metric. Included studies of DIR were used to automatically propagate contour from CT to kVCBCT and vice versa for H&N, pelvis and thorax, but only AI to automatically propagate contour from CT to kVCBCT in H&N satisfied our criteria. Importantly, AI beat DIR with mean DSC score ± SD of DIR v AL (2.44 ± 2.16 mm v 2.71 ± 1.35 mm) for H&N. Mean registration error of DIR for pelvis and lung were 1.79 ± 2.07 mm and 1.32 ± 0.75 mm, respectively.

#### Meta-analysis of the included studies

**Figure 9.**
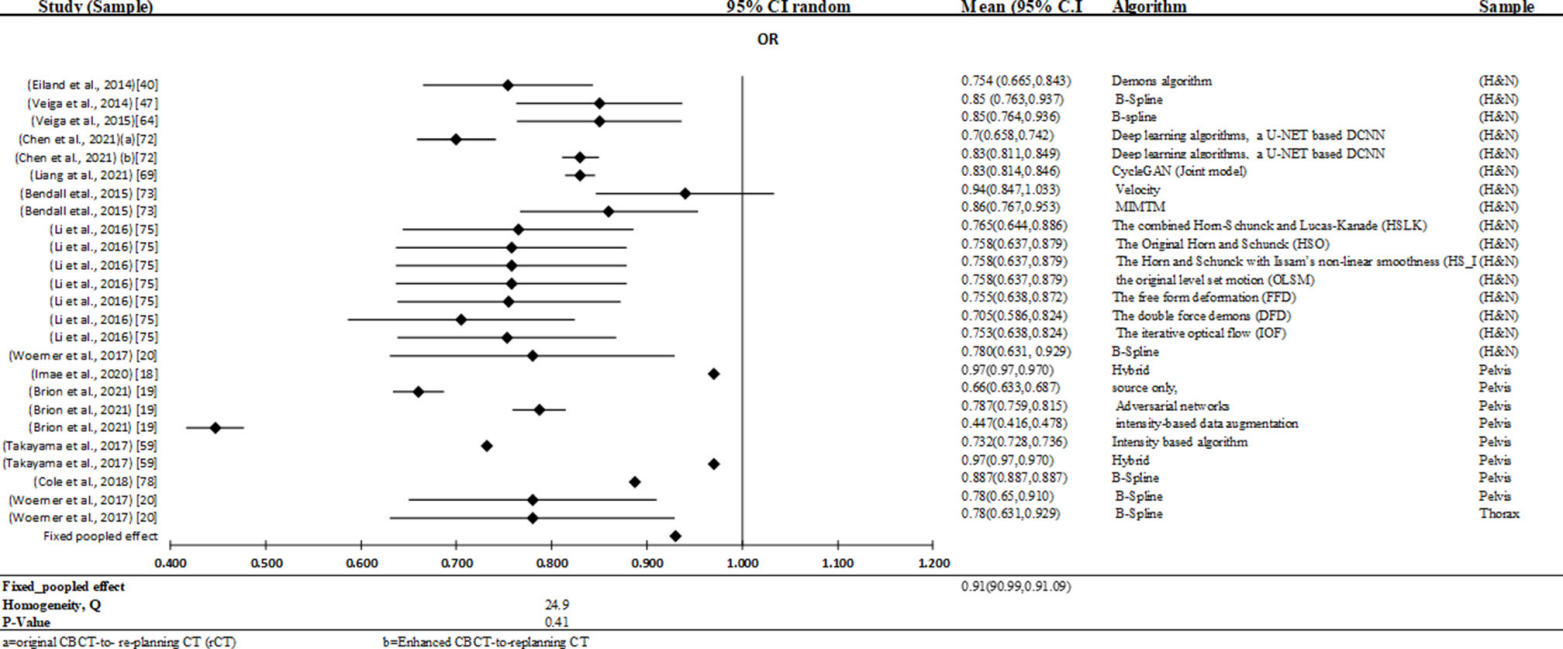
Forest plot of studies that assessed kVCBCT for recontouring accuracy. For automated contouring, the results from the kVCBCT are centered around a DSC of 0.91 with a 95% CI ranging from 90.99% to 91.10%.

In the combined meta-analysis, there were 11 individual articles that presented 25 applications of the segmentation of organs in kVCBCT. The meta-analysis included studies that were evaluated in terms of their DSC. The meta-analysis then indicated a DSC of 0.93 (94.5% CI: 0.9219–0.9301), while the heterogeneity test (Q) showed 24.9 Q-values and 0.41 *P*-values, indicating a lack of significant inhomogeneity among the results, ‘Figure 9’.

The results of individual kVCBCT studies are also shown in ‘Figure 9’. Performance estimation of these studies in terms of the validated DSC rates respectively ranged from 70% (95% CI:65.8–74.2%) to 94% (95% CI: 84.7–100.3%) for H&N, 78% (95% CI:63.1–92.9%) for thorax and 66% (95% CI:63.3–687%) to 97% (95% CI:97–97%) for pelvis, but this depends on the technique used.

### Publication bias

**Figure 10.**
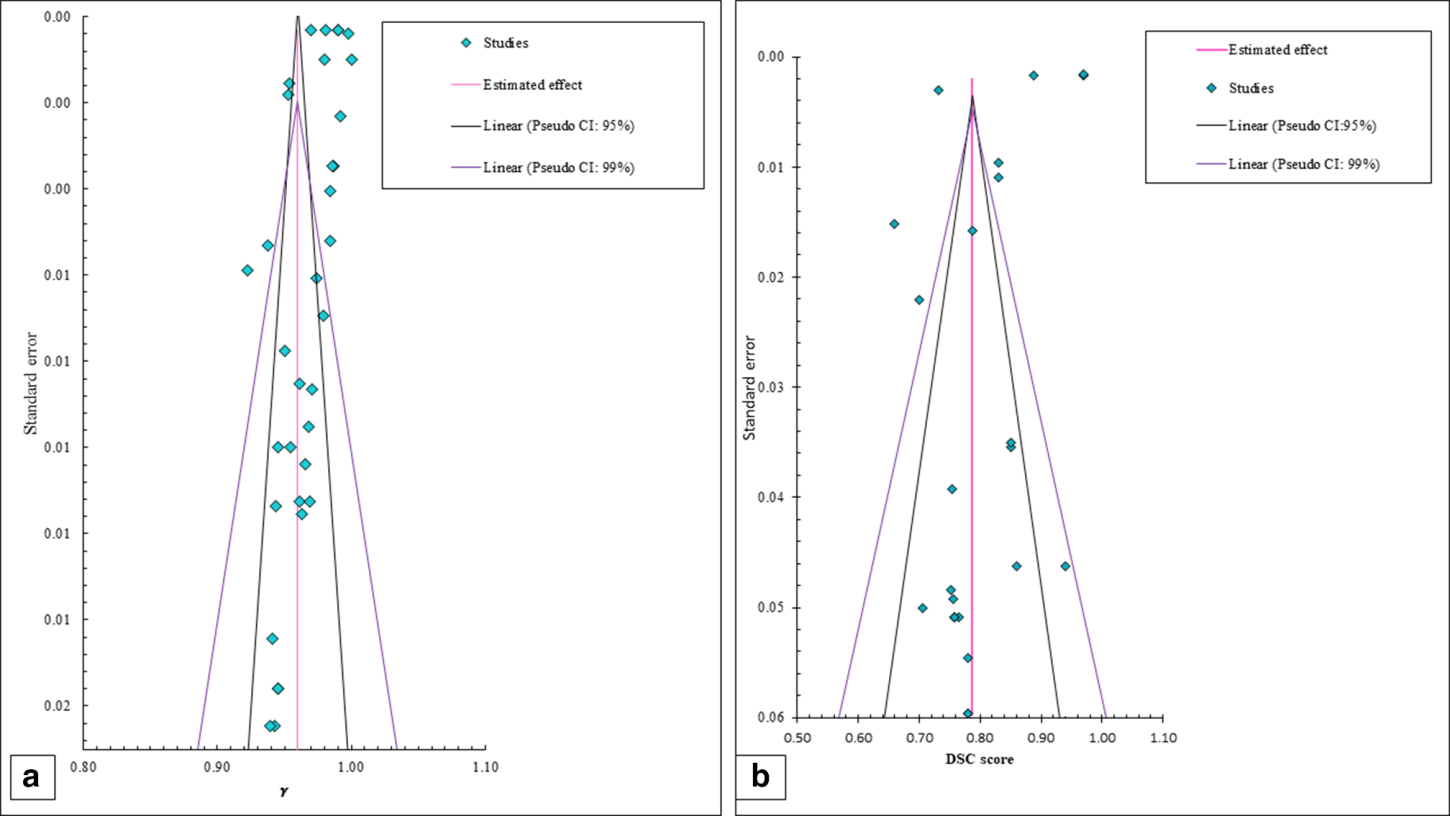
The included studies expressed in funnel plot. (a) Funnel plot for studues that use kVCBCT for contour propagation. (b) Funnel plot for studies that use kVCBCT for contour propagation. Legend: SE, standard error. Horizontal axis represents and DSC as the effect size while vertical axis represents SE.

Funnel plots were schemed using mean γ pass rate and DSC against standard errors for kVCBCT-based dose calculation and kVCBCT-based recontouring to assess potential publication bias, respectively. While applications used in the funnel plot were 36 for kVCBCT-based dose calculation, they were 25 for kVCBCT-based recontouring that were meta-analyzed ‘Figure 10’. An asymmetric funnel plot signaled a bias in publication amongst the included studies. Both kVCBCT-based dose calculation and kVCBCT-based recontouring exhibited significant publication bias. The under curve area of the pseudo-95% CI and pseudo-99% CI did not encompass all studies, which supports the notion of potential publication bias.^84^

## Discussion

kVCBCT has been utilized to compute dose and propagate contours in large numbers of studies examined in this review. H&N, thorax, and prostate cancers have been the subjects of most studies. As a consequence, many studies have suggested that kVCBCT can produce results that mimic those seen in the case of pCT images. However, the comparison has usually not been rigorous. In order to examine this, a meta-analysis of studies on kVCBCT performance in a dose calculation and automated segmentation was necessary.

Currently, resimulated CT is used for dose recalculation and segmentation of tumor lesions for adaptive radiotherapy. Adaptive radiotherapy should be considered in the first instance if it has benefit and if it does no harm in the second instance. However, using resimulated CT is associated with many challenges. Firstly, based on the anatomy shown on CT, patients' physical dose during prostate radiotherapy has been calculated less accurately using dosimetry calculations when compared with kVCBCT. This is because in comparison to true CT images, kVCBCT images have a slightly different anatomic distribution.^85^ Secondly, anatomical changes occur commonly, which dosimetrically requires a new resimulated CT and replanning practice for treatment plan adaptation. This procedure is labor intensive, causes an additional dose to the patient and increases the cost of patient treatment. A pseudo-CT image will only inherit the anatomical information of kVCBCT as long as it has the same anatomy as kVCBCT, and another benefit is the cost saving of not having another CT scan for resimulation. Thus, by replacing current methods (CT and manual segmentation method) with sCT and an automated computer-aided system, improvement of image quality and subsequently treatment replanning can be achieved with small amount of computation time.

This review established that performance of kVCBCT depends on different methods, specific correction method itself and region of interest for dose calculation. For instance, the dose differences between dCT and pCT in H&N were 0.54%, 0.7 and 2.7% for demons, fast demons, and accelerated demons’ algorithms of DIR, but these differences were 0.3%, 1%, 3.9 and 5.4% for PSCC, CC, SSCC and CC, respectively. Similarly, the dose differences between dCT and pCT in pelvis were §amp;gt;2% , ≤2%, and ≤2% for B-Spline, modified Demons and B-Spline algorithms of DIR, but they were ≤ 1.3% for density override and ≤ 0.9 for combined method (DIR+override) while they were 0.6%, 1.2% or 2.3 for CC, respectively. Likewise, the differences between dCT and pCT in thorax were 5.55% and ≤2% or ≤ 5% for Demons and B-Spline algorithms of DIR, but they were 3.9%, 5.4, 1.9 or 2.7% and 2.4 for SSCC, CC, Stoichio-metric calibration and kVCBCT_r_. Importantly, in all included studies recording γ pass rates, pass rates for dose recalculation on corrected images were higher than 90% for all methods, except kVCBCT_LUT_ and kVCBCT_Pop_ that γ pass rates were 85% and 89 ± 8.3. According to this review and meta-analysis of promising results (overall γ pass rate of 98%; 95% CI: 97.79–98.01%), artefact correction methods mentioned above are feasible for dose calculation, but there are many variables encompassed that prevent achieving accepted dose distribution, yet these techniques have a potential capacity to improve the kVCBCT image quality.

On the other hand, this literature also determined that using kVCBCT for automatic contouring depends on propagating method itself, different methods applied and the region of interest for automated contouring. According to registration error mentioned in (Section 3.3), DIR and AI can produce results as good as in the case of registering the same images of the same modality (CT or kVCBCT), registration error of 2.0 mm±5 mm for H&N, but it cannot for pelvis and thorax because they may produce less than 70% DSC and more than 2–3 mm registration error, but the accuracy of contouring depends on the method used. For instance, values of registration error of hybrid registration algorithm and evolution registration algorithm were 0.1 ± 0.6 mm and 0.94–0.3 mm for thorax and pelvis, respectively. Furthermore, regarding the meta-analysis, most of these results suggest that kVCBCT can be used for the segmentation of organs in kVCBCT because volume matching satisfies the recommended value (≥ 70%) for adaptive radiation therapy usages.^37^ The values in 'Figure 9‘ are all higher than 70%, except two applications of studies by (Brion et al.^19^ which scored DSC of o.447 and 0.656 for pelvis using source only ('' A naive strategy for learning to segment target domain images from a dataset of labeled source domain images would be simply to train a neural network on those source images and then test it on test domain images. The kVCBCT from cohort C3 are not used for tests since the images are already used for training the adversarial networks and it is desired to report the performance on an independent test set'') and ''intensity-based data augmentation''.^19^ Therefore, this criterion indicates that the results are sufficient for kVCBCT for automated segmentation provided that appropriate technique for contour propagation is used with satisfactory experience. Morphons, registration algorithms were more accurate than other where velocity and hybrid algorithms scored DSC of 94% (95% CI:84.7–103.3%) and 97% (95% CI:97–97%%) for H&N, and pelvis, respectively. However, for thorax, further scrutinization is required. Notice, kVCBCT showed to become feasible for treatment replanning since that kVCBCT image quality, and subsequently calculated dose, can be improved through artefact correction techniques, and DIR has ability to produce the segmentation similar to that achieved in registering the images of the same modality. Nonetheless, numerous indications of publication bias were seen within this field of study.

sCT images from kVCBCT images can be generated using a number of methods. Using AI has been limited to a small number of included studies. While one study satisfied our criteria was used for dosimetric evaluation, five studies were used for geometric evaluation. Generally speaking, AI algorithms, especially CycleGAN, U-Net GAN and FCN-GAN algorithms, in pelvis were demonstrated that they gave clinically unacceptable dosimetric results, reporting dose differences typically between 3 and 4%. Unsatisfactory results arise from the generator fails to merge deep-layer features and superficial-layer features because there is no a residual network shortcut connection contained in convolutional layers of convolutional neural network-based generator and unavailability of paired data following registration.^54^ On the other hand, for geometric evaluation AI algorithms in H&N demonstrated that they gave clinically tolerable geometric results, recording DSC scores of 0.83 ± 0.06, 0.83 ± 0.09 and 0.92 for U-NET, CycleGAN and DCIGNS, respectively. Nonetheless, AI algorithms in pelvis demonstrated they were heterogeneous. For example, as with U-NET in H&N, one study gave DSC score of 0.79, but another one gave 0.63 in pelvis.^16,19,61,68,83^ This can be attributed to the fact correlation between existence or the non-existence of a given body structure and intensities patterns is taught while prior information of anatomy are unavailable. For example, there is no encouragement to keep away from giving a nil guess for the structure, even although every patient possesses one, when a non-adversarial way is applied to train a network.^19^ However, AI techniques are associated with many benefits in clinical radiotherapy, one of them is resolving the issue of difficult direct modification of an adaptive radiation therapy plan in addition to poor quality of kVCBCT imaging of soft tissues.

For dosimetric evaluation, using DIR for a specific ROI of H&N and pelvis gives satisfactory consequences with overall percentage dose difference of 1% ±1% for H&N and pelvis. Notably, not all DIR algorithms give these delighted results for H&N; for example, when Demons algorithm is solely used in registering of H&N where dose difference was 3%.^39^ However, DIR is disappointing methods for kVCBCT-planning of thorax because overall percentage dose difference was 2% ±2% . The results were out of tolerance (2%) for all registrations techniques and even combined technique (ANIMAL algorithm+HU override), except one study used modified Demons that achieved ≤ 1.9% dose difference.^10,47^ It is dependent on the registration technique used whether the technique can produce an accurate sCT. One of the major drawbacks of DIR-based techniques is their ability to deal with an atypical anatomy. A single DIR by itself has been found to cause geometrical deviations that are unacceptable. DIR techniques showed large errors when patients had surgical voids, larger treatment target or OARs. It should be expected since a single DIR could not deal with the atypical anatomy. Patients with the atypical anatomy should be concerned by the uncertainty in image registration. Many attempts have been made in the literature to avoid this type of problem, for example using hybrid or combined DIR algorithms with or density override or artefact corrected model in the subsequent steps. Importantly, hybrid and combined solutions gave acceptable dosimetric and geometric results in some cases. For instance, γ pass rate of 93.4 ± 6.3 to 99.4 and registration error of ≤ 2 ± 0.1 mm in H&N and DSC score of ≥ 0.8 for pelvis and thorax were achieved.^12,18,41,51,59,75^

HU override-based techniques are encouraging methods for kVCBCT-only dose calculation. Routine kVCBCT can be used and the process can be entirely automated^1^. The techniques can be achieved using a single in-room kVCBCT system, precluding requirement for resimulated CT and reducing probabilities of patient movement. This method has been shown to yield results with adequate dosimetric accuracy for H&N, thorax and prostate, with dose deviation ≤ 1.9%.^9,43,66,80^ However, one study reported using overrides for the cancer case of patient prostate could give unacceptable dosimetric outcomes, with dose deviation of 3.2%. A vendor-specific variation in kVCBCT image quality accounts for this issue; in turn, this could impact the accuracy of override-based technique. For radiotherapy departments to identify the best use of override-based technique for their resources, similar investigations should be subsequently carried out using their kVCBCT image databases.^10^ One of the major disadvantages of override-based is the techniques show to produce results with low level of image quality, particularly when step of override is automated. As a result, automatic segmentation of different tissue classes produces results with unacceptable outcomes. It is likely that combined methods are used in the subsequent correction steps to resolve this issue, such as DIR with override-based technique for dose calculation, and then different tissue classes are robotically segmented using DIR.^41^

Calibration curve-based strategies have been showed up to provide clinically heterogeneous dosimetric outcomes, which typically depend on the method used and ROI. For instance, Dose differences are typically lower than 2% have been reported when patient-specific calibration or HU-ED calibration, stoichiometric calibration or HU-ED calibration and standard calibration curves are used for H&N, thorax, and pelvis, respectively. Hence, creating accurate reference images for dose guidance has proven feasible in these ways.^10,42,50,57^ In contrast, dose differences of more than 2% were reported when using standard calibration curves and HU-ED calibrations for H & N and pelvis, respectively.^10,38,43,53,57,58^ One of the main drawbacks of calibration curve-based method is that this technique has been shown to produce low quality results. Thus, automatic segmentation of various tissue classes produces unacceptable results. Subsequently, correction steps to solve this problem, such as DIR with calibration curve technique for dose calculation are applied, and then classes of different structures are automatically segmented using DIR.^52^

It must be justified why any metric is used. However, the gold standard adopted should be γ analysis with clinical and statistical parameters to avoid challenges and shortcomings in dose calculation.^86^ Included studies in this review often adopted DVH and γ analysis for methodologies of dose comparison for treatment plan verification. Although some included studies gave critical parameters of these matrices such as reference dose, threshold, DTA and, type of γ analysis (local or global) and dimension (2D or 3D), but they were heterogenous in several studies while other studies missed these parameters. This may be a reason of publication bias within this field of study in addition to no awareness in the publication of unwell performing artefact correction or propagation methods. This problem has to be solved by using γ analysis with consistent reporting of clinical and statistical parameters and DVH parameters for ROI in patients.

The current meta-analysis of kVCBCT for dose recalculation and automatic segmentation has a number of methodological shortcomings. Firstly, currently, a common ground truth does not exist for reference doses. Usually, authors use CT as ground truth to minimize the contribution of geometrical changes, but CT images have a slightly different anatomic distribution and suffer from non-constant image quality due to variability of image acquisition parameters.^85^ Secondly, a variety of variables, such as the TPS algorithm, the tumor position, the acquisition of images, etc., affect the dose difference evaluation. Thirdly, there was considerable heterogeneity among analyses, likely because of technical differences between correction methods for segmentation. Finally, missing data resulted in excluding of several studies.

Studies have been put on the individual cases rather than population so far. Most of these studies were to evaluate the capability of image correction algorithms and models. Subsequently, many studies used few numbers of patients to test their approaches (less than ten patients). Therefore, it is noticeable that patients with untypical anatomy is inconceivable to become sufficiently tested and clinical potentiality is scarcely illustrated although this type of studies may possibly display prototype. In conclusion, a large patient cohort of clinical studies to evaluate kVCBCT-based treatment replanning is undoubtedly required.

Although kVCBCT performing kVCBCT -based dose calculation and automated segmentation have shown fairly promising results (overall γ pass rate score of 0.93; 95% CI: 0.9299–0.9399 and overall DSC value of 0.98; 95% CI: 0.9799–0.9801), this methodology is still not widely accepted and employed in daily clinical practice. This may be explained by many reasons. Firstly, standardized procedures to accurately use these correction and segmentation systems are not available. The current standards of adaptive radiotherapy and a state-of-the-art system that automatically propagates contours and scan from CT to kVCBCT are substantially different, which subsequently obstructs addition of correction methods. Secondly, the new innovation is to use kVCBCT for dose calculation and automated segmentation, but the traditional purpose for the kVCBCT use varies; where radiographers mainly use this technology for setup by comparing the pCT(or reference images) to CBCT before treatment delivery to check the positioning of the patient. This ensures reproducibility of patient set up during the course of radiotherapy treatment. Furthermore, implementation of kVCBCT methods in radiation oncology requires interaction between clinician and computer equipped with high sophisticated technology. Subsequently, trained observers are needed to supervise framework of dose calculation and automated segmentations. Moreover, there are different artefact correction and segmentation methodologies with different details that have a different effect on the consequences in applications of the same dataset. For instance, in RayStation TPS, there are intensity-based algorithm and hybrid (biomechanical model+intensity-based algorithm), but in DIRART, the top registration algorithms are optical flow and modified demons.^12,13^ kVCBCT can back the adaptive radiotherapy up without the need of resimulated CT. Thus, the future application of kVCBCT in the treatment replanning is of great clinical consequence. More remarkably, sCT is highly likely to form the basis of further cutting-edge analyses to illuminate reliable and meaningful relations between features-based kVCBCT imaging and survival rate, for example kVCBCT-based dose accumulation and Prediction of radiation response of tumors using kVCBCT-based radiomics.

## Conclusion

A systematic review and meta-analysis of various studies using kVCBCT for treatment replanning were performed to identify how kVCBCT can be used for kVCBCT-based treatment replanning performance. DIR-based method has been recognized as the most clinically valuable. This is because it can correct kVCBCT for dose calculation and contour propagation with high level of accuracy. Most of studies used in-house specific techniques, yet radiotherapy community starts becoming interested. Therefore, commercial techniques have been developed and became available. Since there is growing appeal of kVCBCT-based adaptive radiotherapy, large cohorts of patients have to be undertaken to authenticate methods within a kVCBCT-based adaptive radiotherapy workflow. Since hybrid registration method gives good outcomes of contour propagation in challenging cases, namely pelvis, these cohorts studies should focus on using hybrid registration method for different spectrum of patients with H&N, thorax, and pelvis cancers.^12,18,75^ Importantly, they should focus on both dose calculation and contour propagation. Additionally, small numbers of included studies dealt with AI; therefore, further studies focusing on comparing AI to hybrid registration method-based DIR in terms of both dose calculation and contour propagation using AI for different spectrum of patients are also required. Quality guidelines should be followed when reporting on kVCBCT, which includes agreed metrics for reporting on the quality of corrected kVCBCT and validation on a set of external tests. Also, recovering quality of kVCBCT image can enhance dose calculation and segmentation results, so it is recommended to define protocols of new site-specific standardized imaging that can be used when obtaining kVCBCT images for treatment replanning.
